# From accurate genome sequence to biotechnological application: The thermophile *Mycolicibacterium hassiacum* as experimental model

**DOI:** 10.1111/1751-7915.14290

**Published:** 2023-07-27

**Authors:** Mercedes Sánchez‐Costa, Susanne Gola, Marta Rodríguez‐Sáiz, José‐Luis Barredo, Aurelio Hidalgo, José Berenguer

**Affiliations:** ^1^ Department of Molecular Biology, Faculty of Sciences Centre for Molecular Biology (UAM‐CSIC) University Institute for Molecular Biology Madrid Spain; ^2^ Department of Medical Technology and Biotechnology Ernst‐Abbe‐Hochschule Jena, University of Applied Sciences Jena Germany; ^3^ Department of Biotechnology Curia, Parque Tecnológico de León León Spain

## Abstract

Mycobacteria constitute a large group of microorganisms belonging to the phylum *Actinobacteria* encompassing some of the most relevant pathogenic bacteria and many saprophytic isolates that share a unique and complex cell envelope. Also unique to this group is the extensive capability to use and synthesize sterols, a class of molecules that include active signalling compounds of pharmaceutical use. However, few mycobacterial species and strains have been established as laboratory models to date, *Mycolicibacterium smegmatis* mc^2^155 being the most common one. In this work, we focus on the use of a thermophilic mycobacterium, *Mycolicibacterium hassiacum*, which grows optimally above 50°C, as an emerging experimental model valid to extend our general knowledge of mycobacterial biology as well as for application purposes. To that end, accurate genomic sequences are key for gene mining, the study of pathogenicity or lack thereof and the potential for gene transfer. The combination of long‐ and short‐massive sequencing technologies is strictly necessary to remove biases caused by errors specific to long‐reads technology. By doing so in *M. hassiacum*, we obtained from the curated genome clues regarding the genetic manipulation potential of this microorganism from the presence of insertion sequences, CRISPR‐Cas, type VII ESX secretion systems, as well as lack of plasmids. Finally, as a proof of concept of the applicability of *M. hassiacum* as a laboratory and industrial model, we used this high‐quality genome of *M. hassiacum* to successfully knockout a gene involved in the use of phytosterols as source of carbon and energy, using an improved gene cassette for thermostable selection and a transformation protocol at high temperature.

## Graphical abstract

- *Mycolicibacterium hassiacum*, the most thermophilic mycobacterium so far studied, is able to use phytosterols as carbon source.
- A curated version of the genome combining long and short read sequencing technologies was used to identify genes involved in sterols metabolism.
- The first marker for thermostable selection in thermophilic mycobacteria has been developed and used for the directed isolation of mutants defective in phytosterols degradation.

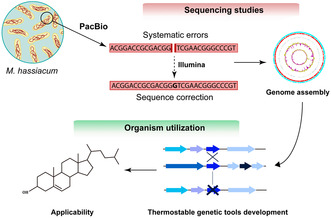

## INTRODUCTION

Until its recent division in five genera (Gupta et al., 2018), the genus *Mycobacterium* comprised approx. 190 species, many of them relevant pathogens for humans or animals, including the causative agent of a still very relevant disease such as tuberculosis, *Mycobacterium tuberculosis*.

For practical reasons, classical taxonomy divided the mycobacteria into slow‐ and fast‐growing strains (Runyion, 1959), the latter being those that form colonies on plates in less than 7 days, whereas most of the pathogenic ones are slow‐growing. In fact, faster growth was a main driver to the adoption of the fast‐growing non‐pathogenic species *Mycolicibacterium smegmatis* (homotypic synonym: *Mycobacterium smegmatis*) as a proxy model for slow‐growing pathogens like *M. tuberculosis*.

The cell envelope of mycobacteria is complex, with several layers including a highly hydrophobic outer membrane. This “mycomembrane” is made of long‐chain 2‐alkyl 3‐hydroxy fatty acids, known as mycolic acids, with some external lipids at the outer face, resulting in low permeability to many compounds (Dulberger et al., 2020; Marrakchi et al., 2014; Zuber et al., 2008). The synthesis of mycolic acids has been used as target for antibiotic therapy against infections by pathogens of the genus leading to approval of the drugs isoniazid (INH), ethionamide (ETH), isoxyl (ISO) and thiacetazone (TAC) (North et al., 2014). Mycolic acids are bound to a Gram‐positive‐like peptidoglycan cell wall through polysaccharide chains (arabinogalactans). Underlying the cell wall, the cytoplasmic membrane is also particular as it contains relevant amounts of sterols, whose biosynthetic capacity is encoded in many strains of the family. In fact, the presence of pathways for the production and modifications of sterols in mycobacteria is of biotechnological interest for the bioconversion of the sterol structures to produce relevant drugs for the pharmaceutical industry (Galan et al., 2017; Xiong et al., 2020).

For this purpose, modified model mycobacteria such as *M. smegmatis* can be used in fermentation processes to allow the accumulation of intermediates of interest from cheap steroid precursors such as plant phytosterols (Galan et al., 2017; Garcia‐Fernandez et al., 2017; Josefsen et al., 2017; Xiong et al., 2020). However, most models and genetically tractable mycobacteria grow at temperatures below 35°C, at which the solubility of steroid substrates and products is limited (i.e. 0.095 mg/L for cholesterol at 30°C; or 40 mg/L for Androstenedione at 37°C; https://pubchem.ncbi.nlm.nih.gov/compound/5997; /compound/6128).

A main challenge in the conversion of phytosterols to steroid drug precursors is that both substrates and products show very little solubility in water, making the use of two phases media and/or solubilizing agents such as cyclodextrins unavoidable (Josefsen et al., 2017). A good strategy to increase the solubility of substrates and products and their rate exchange is to increase the temperature of the reactions. However, thermophilicity is a rare property among mycobacteria, with *Mycolicibacterium hassiacum* (homotypic synonym: *Mycobacterium hassiacum*) being the most thermophilic species isolated so far (Schroder et al., 1997; Tortoli et al., 1998). Despite being originally isolated from a human urine sample, *M. hassiacum* grows very slowly at 37°C, increasing its growth rate to reach a maximum around 55°C and having still significant growth capability up to 65°C. The strain is also quite tolerant to salt (up to 5% (w/v) of NaCl) and is unable to use most sugars or citrate as carbon sources (Schroder et al., 1997).

In this work, we analysed a curated whole genome sequence (Sanchez et al., 2019) in search for clues to assess the potential of *M. hassiacum* to acquire DNA through the identification of HGT‐related properties such as the presence of type VII secretion systems likely involved in conjugation, CRISPR‐Cas clusters and insertion sequences. Then, we showed the ability of this strain to degrade phytosterols and used the curated genome to reconstruct a hypothetical phytosterol degradation pathway. In this hypothetical pathway, we identified a putative gene encoding a homologue to 3‐ketoxosteroid delta1‐dehydrogenase (*kstD4*), as a likely target for the isolation of knockout mutants that could accumulate intermediates of the pathway. Finally, we modified the growth conditions of *M. hassiacum* to allow its transformation and developed a synthetic gene cassette conferring thermostable resistance to Kanamycin for the isolation of the corresponding insertional knockout mutant by homologous recombination. To the best of our knowledge, this is the first report in which a thermophilic mycobacterium is subjected to directed genetic manipulations, opening new avenues for the use of *M. hassiacum* as a laboratory model and for biotechnological purposes.

## EXPERIMENTAL PROCEDURES

### Strains and growth conditions

*M. hassiacum* was acquired from the German Collection of Microorganisms and Cell Cultures (DMSZ) under the code DSM 44199. Routinely, *M. hassiacum* was grown at 50°C in an orbital shaker at 180–200 rpm. For liquid cultures, complete Middlebrook 7H9 broth with glycerol as the carbon source and supplemented with 0.15% (v/v) Tween® 80 and 10% (v/v) OADC (supplement for Mycobacteria growth medium composed of oleic acid, bovine albumin, glucose, catalase and NaCl, https://www.sigmaaldrich.com/ES/es/product/sial/m0678) was used. Growth in solid media was carried out in Middlebrook 7H10 also supplemented with 10% (v/v) OADC. Selection with kanamycin (20 μg/mL) was employed when required.

To analyse the antibiotic susceptibility, agar plates of GPHF‐medium plates (DSMZ 553, https://www.dsmz.de/microorganisms/medium/pdf/DSMZ_Medium553.pdf) were used (Composition: 0.5% (w/v) beef extract, 0.5% (w/v) pancreatic digestion of casein, 0.5% (w/v) yeast extract, 0.073% (w/v), 1.5% (w/v) agar with 1% (w/v) glucose and 0.8% (w/v) glycerol). For this, 100 μL of serial dilutions of *M. hassiacum* grown until saturation in GPHF broth (same composition without agar) were spread on plates. Antibiotic discs were prepared by impregnating filter paper with 10 μL of Hygromycin B (Hyg), Kanamycin (Kan) and Streptomycin (Str) at 5, 10, 25, 100, 200 and 500 ng/μL. Plates were incubated at 55°C for 48 h.

### Optical and electron microscopy

Cells for transmission electron microscopy were re‐suspended in MQ water and negatively stained with 2% (w/v) uranyl acetate. A collodion grid of 400 mesh with carbon shadow was used as support. The microscope used was a JEM 1010 (Jeol) with a CMOS TemCam F416 (TVIPS) camera. Phase contrast optical microscopy images were acquired with an Olympus BX61 microscope coupled to a Hamamatsu C7780 camera.

### Genome analysis

The curated genome of *M. hassiacum* used (Sanchez et al., 2019) was obtained by combining the reads obtained by SMRT (single‐molecule real time) of PacBio Biosciences and those previously obtained in a sequencing project of the Joint Genome Institute (https://genome.jgi.doe.gov/portal/pages/projectStatus.jsf?db=MycDSM44199_FD) with Illumina technology (https://www.ebi.ac.uk/ena/browser/view/SRR3947910?show=reads). The analysis of the genetic context around the systematic sequence errors found when compared both sequencing technologies was carried out by an *in house* script written in Perl to initially determine the GC content, and MEME Suite to detect overrepresented motifs (Bailey et al., 2009). CRISPR Web Server, CRISPRminer and IS finder were employed to identify CRISPR and IS sequences. The identification of genes putatively involved in the sterols metabolism was done through the KEGG pathway server, and further refined through BlastP analysis and manual inspection. The multi alignment of amino acid sequences was performed with Clustal Omega program. The molecular dynamics and docking simulations to construct the 3D models of the candidate enzymes were carried out by NAMD and AutoDock softwares, respectively. The PyMOL Molecular Graphics System, Version 2.0 (Schrödinger, LLC) was used to generate the images of KstD models from *Sterolibacterium denitrificans* and *M. hassiacum*.

### Plasmids used

All the plasmids used in this work are described in Supplementary Table 1. Integrative vectors pMY769 (Forti et al., 2009), pMC1S (Guo et al., 2007) and pTTP1B (Pham et al., 2007) and the replicative vector pJV53 (van Kessel & Hatfull, 2007) were used as candidate vectors in *M. hassiacum*. The pMH3 plasmid was generated from pUC19::*kat* backbone (Blesa et al., 2017) by replacing the P*slpA* promoter of the *kat* cassette encoding a thermostable resistance to kanamycin by the promoter that controls the kanamycin resistance gene in pTTP1B (Guo et al., 2007). For this, the promoter was PCR amplified from pTTP1B with primers Prom_pTTP1B_Fw (5′TTTGACGTCTCCTGGTATGCAGCCT3′) and Prom_pTTP1B_Rv (5′CATATGAACACCCCTTGTATTACT3′) and integrated at the *Aat*II and *Nde*I restriction sites (underlined in primer sequences) of the pUC19::*kat* plasmid to obtain plasmid pMH3 (Supplementary Table 2).

### Transformation and isolation of insertion mutants

Electroporation of *M. hassiacum* was achieved by the method described by (Goude et al., 2015) with modifications. Culture media and growth conditions were chosen to obtain efficient growth rates and limited cell aggregation in the cultures. The best conditions selected were 7H9 medium with 10% (v/v) OADC and 0.15% (v/v) Tween® 80, at 50°C under 200 rpm rotary shaking. Electrocompetent cells were obtained from cultures grown under these conditions up to an OD_600_ of 0.8–1. The cultures were cooled on ice for 1 h, and cells were harvested by centrifugation for 30 min at 3000× *g* at 4°C. The cells pellet was re‐suspended and washed four times with pre‐chilled 10% (v/v) glycerol, reducing 1/2 of the volume each time. Cells were finally re‐suspended in 1/50 of the original volume in the same glycerol solution, and aliquots were flash frozen in liquid nitrogen for storage at −80°C. For transformation, 200 μL aliquots were thawed on ice, transferred to 0.2 cm Bio‐Rad Gene Pulser cuvettes and incubated for 15 min at 4°C with 500 ng of the corresponding plasmid (purified by Thermo Scientific GeneJET plasmid miniprep kit). The electric pulse was carried out in a 0.2 cm wide cuvette with an Equibio, Easy Plus 2000 device (2.5 kV, 200 Ω and 25 F, pulse time below 5 ms). 5 mL of preheated (42°C) complete 7H9 broth were added to the cells for subsequent incubation at 42°C for 7 h. Afterwards, cells were harvested by centrifugation at 3000× *g* at room temperature for 15 min, carefully re‐suspended in 200 μL of culture media and spread on 7H10 agar plates with the corresponding selection antibiotic. Plates were incubated at 42°C as a compromise between growth rate and the expectable lability of the enzymes (i.e. replication, integration and antibiotic modification) encoded by the plasmids, all of them from mesophilic origin. A wet chamber was used to limit evaporation during the 7‐days incubation period.

For the generation of *kstD4* mutants by a single homologous recombination event, an internal region of the gene was amplified with primers kstD_Fw (5′AAACTCGAGATGCTGTCGTTCGTCAT3′) and kstD_Rv (5′TTTCTCGAGGATCACCGATCCGTCGT3′) and cloned at *Sal*I restriction site of pMH3 (underlined), to obtain plasmid pMH3::*kstD4*. This plasmid was further used to transform *M. hassiacum*.

### Biotransformation assays

Sterol biotransformation experiments were performed in a medium composed of 0.5% L‐asparagine; 0.59% KH_2_PO_4_; 0.5% K_2_SO_4_; 0.15% citric acid and 0.06% MgSO_4_ (w/v), supplemented with 0.15% (v/v) Tween® 80 and, at least, 0.1 g/L of phytosterols as the major carbon source. Solutions of dispersed phytosterols were prepared by adding different amounts of soya phytosterols extract (Advasterol™ 90, from Advanced Organic Materials) to distilled water containing 1.5% (v/v) Tween® 80 and subjecting the mix to sonication for 15 min at 100% amplitude using an ultrasonic probe homogenizer (Labsonic®). Finally, the solution was sterilized at 121°C, for 15 min and added at 50°C to the sterile growth media to generate the biotransformation medium (BM). The inoculated BM was kept for up to nine days at 50°C in an orbital shaker at 180–200 rpm.

### Analysis of phytosterols bioconversion

Thin‐layer chromatography (TLC). Samples from cultures grown with phystosterols, including the corresponding cells, were extracted with 10× volumes of ethyl acetate and, after vigorous shaking, phases were separated by centrifugation. Components from the organic phase were then separated in TLC sheets (Silica gel 60 F524, Merck). Phytosterols from soya and androst‐4‐ene‐3,17‐dione (androstenedione, AD) at 1 and 10 ng/μL in ethyl acetate, respectively, were employed as standards. A mixture of hexane: ethyl acetate 1:1 (v/v) was used as mobile phase, and plates were developed with 20% (v/v) sulphuric acid in water and incubation at 100°C until spots were sufficiently visible.

High‐performance liquid chromatography (HPLC). Samples from the biotransformation media were extracted with one volume of ethyl acetate and examined by HPLC, using a 250 × 4.6 mm Nucleosil C‐18 column with 5 μm particle size. The mobile phase was 52:48 (v/v) acetonitrile: water with 0.01% (v/v) of acetic acid, and the separation was performed at 50°C for 30 min. The absorption of each peak was monitored by optical spectrometry. AD and 1,4‐androstadiene‐3,17‐dione (androstadienedione, ADD) were used as standards.

Ultra‐performance liquid chromatography‐mass spectroscopy (UPLC‐MS). Samples from the biotransformation media were extracted with one volume of methanol and analysed by UPLC‐MS with a PDA photodiode array detector coupled to an ESI‐TOF (time‐of‐flight mass spectrometer) LCT Premier XE from Waters. The column was a C18 of 50 × 2.1 mm and particle size of 1.7 μm. The elution buffers were (A) 2% acetonitrile and 0.1% formic acid (v/v) and (B) methanol and 0.1% formic acid (v/v) and the gradient employed was 5%–99% of (B). The MS was set up from 50 to 500 m/z. Data analysis was done in Masslynx version 4.1. Phytosterols were detected by absorbance at 243 nm.

## RESULTS AND DISCUSSION

### The curated genome of *M. hassiacum*

The assembled genome of *M. hassiacum* into a single circular chromosome was achieved previously (Sanchez et al., 2019) by combining long‐reads sequencing (Pacific Biosciences) and a partial genome of the same strain obtained within a JGI project by Illumina technology (https://www.ebi.ac.uk/ena/browser/view/SRR3947910?show=reads). The comparison between the independent assemblies obtained with both technologies revealed apparent frameshift mutations in tracks of G or C at 483 positions in the genome (Sanchez et al., 2019), similar to those further detected in the sequence of *Thermus thermophilus* NAR1 (Sanchez‐Costa et al., 2020).

**FIGURE 1:**
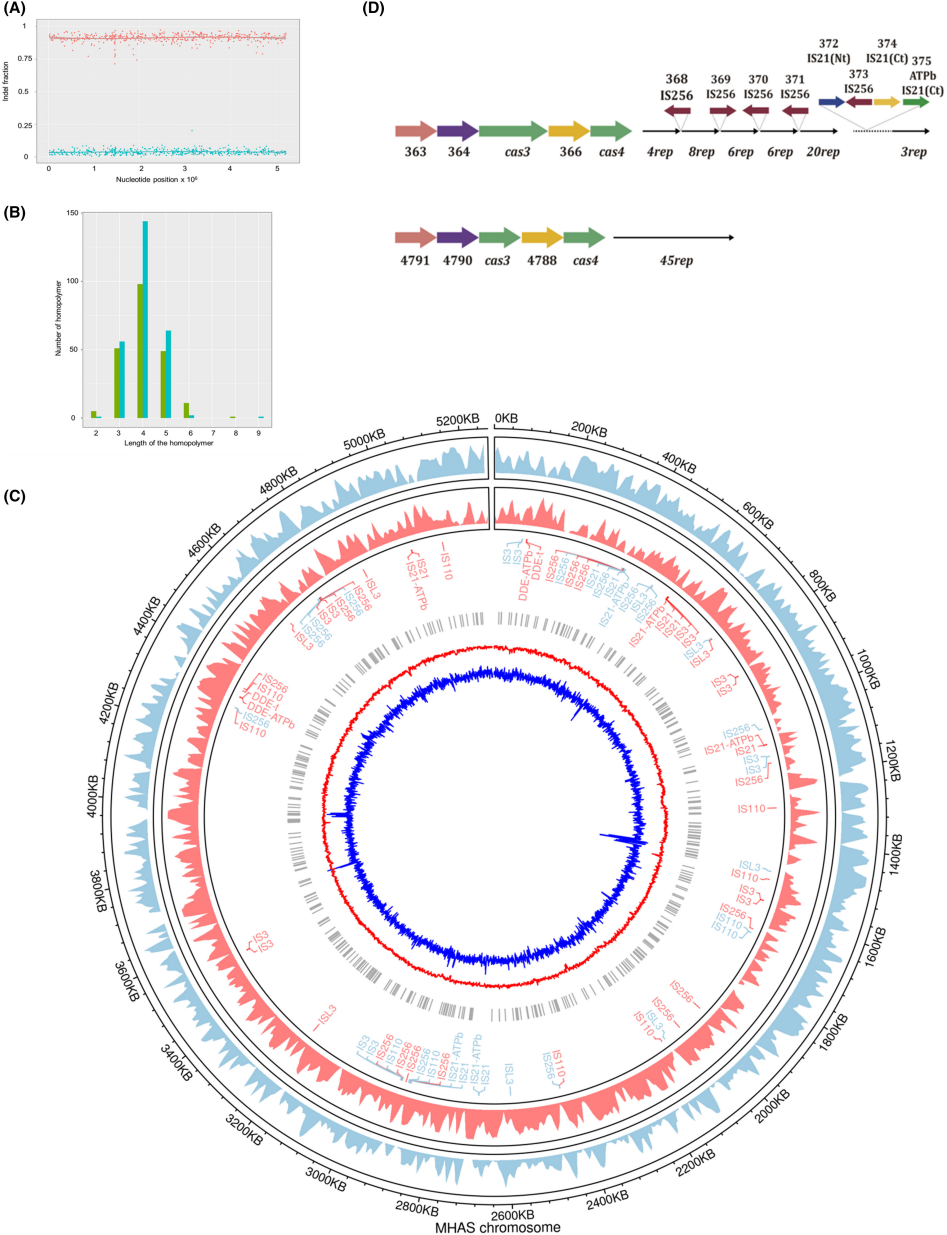
Curation and analysis of the genome of *Mycolicibacterium hassiacum*. (A) Figure shows the fraction of the reads by Illumina (red dots) that contained a given base compared to the ratio of PacBio reads (blue dots) in which this base appeared. A trend line crosses the points distribution. (B) Distribution of the errors detected in (A) when comparing Illumina and PacBio reads of the *M. hassiacum* sequence. Green colours correspond to C tracks and blue colours corresponds to G tracks. Note that the number of homopolymers in the genome are 34770, 7021 and 548 for the 4, 5 and 6 G/C tracks, respectively. (C) The chromosome of *M. hassiacum*. Numbers indicate the position in the genome. From outside in: Thick marks designate ORFs from the forward (blue) and reverse (red) strands. Next, the position of ISs marked in blue or red, depending on the position in the forward or reverse strand, respectively. Grey vertical marks show the position of the G/C errors detected in the comparison with the short reads sequencing with Illumina. The inner red circle corresponds to GC content), and the inner circle in dark blue indicates the GC skew which tells the degree to which the GC content is skewed toward G or skewed toward C. (D) CRISPR clusters in *M. hassiacum*. Figure represent the ORFs numbers (colour arrows) of the genes encoding the two CRISPR clusters detected in the genome of *M. hassiacum* in which homologues to Cas4 and Cas3 have been identified. ISs are identified along the coding number in the *M. hassiacum* genome.

In order to know how frequent these differences between both technologies were, the original reads of both technologies were compared at these 483 positions in the genome. As shown in Figure 1A, 90% of the PacBio reads missed 1 bp at each of these positions (except for two positions missing 2 and 3 bases) respect to the 95% of the Illumina reads in which this base was included. Most of these systematic errors were located at 4G/C homopolymers (Figure 1B), but they still represent only the 0.68% of all the 4G/C tracks of the genome, suggesting some kind of specificity in the reading errors. This percentage increased slightly with the size of the G/C homopolymers, to 1.45% and 2.2% for those of 5 and 6 bp, respectively.

A search for differences in G/C content or for the presence of conserved sequence features 20 bases upstream and downstream of the specific misread tracks did not reveal any proximal sequence signature that could explain such error specificity at these tracks and not at others (Supplementary Figure 1). However, the putative existence of longer range structural effects that could influence the processivity or favour the slippage of the sequencing enzymes cannot be disregarded by our analysis.

Once these systematic errors were corrected, the curated genome of *M. hassiacum* has a full size of 5,269,097 bp, encoded within a single circular chromosome (Figure 1C). This size is intermediate between those from pathogenic mycobacteria, such as *M. tuberculosis* (4.4 Mbp) or *Mycobacterium avium* (4.83 Mbp) and those of saprophytic ones such as the laboratory model *M. smegmatis* (6.46 Mbp) or *Mycobacterium fortuitum* (6.35 Mbp). The absence of Illumina reads not matching the chromosome obtained by PacBio assemble supports the fact that *M. hassiacum* DSM 44199 does not contain plasmids, in contrast to other species of the *Mycolicibacterium* genus (Morgado & Vicente, 2021).

Functional annotation and grouping of the 4979 ORFs detected into KEGG pathways allowed the assignation of 1224 of the genes to 231 pathways, some of them including more than 10 genes (Supplementary Figure 2).

### Insertion sequences and CRISPR‐Cas in *M. hassiacum*

In order to know the susceptibility of this strain to acquire DNA from the environment, from phages or from other mycobacteria, a search for genes indicative of lateral transfer was carried out. Among the detected genes, the relevant number and diversity of insertion sequences (ISs) (Table 1) constitute a major indicator of the ability of the strain to receive foreign DNA (Touchon & Rocha, 2007). Based on blast analysis, some of these ISs encoded complete transposases, whereas other copies contained truncated ones (Table 2). Among the ISs, there are 17 complete and 8 incomplete copies of a transposase belonging to the IS256 family, 9 complete and 2 incomplete copies of IS110‐like transposases and 9 complete copies of an ISL3‐like insertion sequence.

**TABLE 1:** Distribution of ISs on the *Mycolicibacterium hassiacum* chromosome.

IS family	Genome start position (bp)
IS110	1,294,097; 1,511,194; 1,680,643; 1,681,299; 2,106,054; 2,442,951; 2,897,773; 2,921,245; 4,295,929; 4,351,716; 5,122,272
IS21	405,533; 408,050; 558,910; 559,670; 1,101,517; 2,686,862; 2,892,945; 5,027,620
IS256	396,558; 398,704; 400,530; 402,427; 406,669; 500,419; 503,019; 1,053,476; 1,157,794; 1,667,494; 1,955,155; 2,038,553; 2,444,414; 2,896,931; 2,900,956; 2,907,621; 2,920,403; 2,923,263; 4,297,392; 4,359,525; 4,709,310; 4,713,667; 4,718,534; 4,719,692; 4,720,973
IS3	85,462; 85,767; 560,662; 561,333; 878,055; 878,912; 1,138,429; 1,138,734; 1,576,715; 1,577,572; 2,925,868; 2,926,173; 3,540,894; 3,541,751; 4,715,895; 4,716,752
ISL3	501,440; 739,282; 740,964; 1,487,374; 2,096,180; 2,592,895; 3,224,583; 4,600,526; 4,871,301
DDE‐t (IS21 family)	98,630; 4,331,611
IS21 ATPb	408,654; 558,111; 1,100,718; 2,688,283; 2,894,569; 5,026,821
DDE‐ATPb	97,776; 4,330,757

**TABLE 2:** Presence of ISs in the genome of *Mycolicibacterium hassiacum.* From left to right columns indicate the belonging family, number of complete ISs and their size, number of incomplete ISs including N‐terminal or C‐terminal region and their size, respectively, the score obtaining in the alignment and the organisms containing ISs with higher homology.

Family	Compl. (bp)	N‐t (bp)	C‐t (bp)	Score	Closer organism
IS110	9 (1230)	1 (660)	1 (573)	0.0	*M. thermoresistible, Mycobacterim palauense*
IS21	4 (1623)	2 (867–1047)	2 (603–795)	0.0	*M. thermoresistible*, *M. phocaium, Rhodococcus rhodochrous*
IS256	17 (1320)	2 (375–933)	6 (609)	0.0	*M. smegmatis*, *M. chlorophenolicum*, *Mycobacterium avium*, *Gordonia* spp.
IS3	—	8 (309)	8 (861)	0.0 (C‐t)	*M. smegmatis*, *Mycobacterium abscessus*
				5·10^−68^(N‐t)	
ISL3	9 (1329)	—	—	0.0	*M. thermoresistible*, *M. frederiksbergense*, *Mycobacterium kansasii*, *Rhodococcus subtropicis*
DDE‐t (IS21 family)	2 (1362)	—	—	0.0	*Mycobacterium frederiksbergense*, *Mycobacterium europaeum*
IS21‐ATPb	6 (798)	—	—	0.0	*M. thermoresistible*
DDE‐ATPb	2 (858)	—	—	0.0	*Mycobacterium pseudokansasii*

In addition to these well‐conserved ISs, it is also relevant to note the presence of copies of genes coding for putative ATP‐binding proteins close to the 3′ terminus of IS21‐like transposases and to copies of a gene coding for a putative DDE‐transposase (IS‐like). It is tempting to speculate on putative helper enzymes similar to those present in complex ISs, where accessory genes of unknown function are located (Siguier et al., 2014). The presence of eight copies in the genome of two linked sequences encoding homologues to the N‐terminal and the C‐terminal domains of an IS3‐like insertion sequence is also noteworthy. This conservation of N‐ and C‐terminal transposase‐domain homologues among these eight copies suggests an active joint mobilization capability likely through ribosomal re‐adjustment of the reading frame to produce an entire protein, as described for many members of the IS3 family (Sekine & Ohtsubo, 1989; Siguier et al., 2014). In total, IS and IS‐like sequences account for the 1.49% of the genome size, a figure similar to that found in other mycobacteria.

As shown in Table 2, sequences identical to all these putative mobile elements can be found in the genomes of other species of the genera *Mycolicibacterium*, *Mycobacterium* or *Rhodococcus* (Table 2), supporting the existence of recent genetic exchange between strains of these three genera.

Interestingly, many ISs were found inside one of the two CRISPR clusters present in the genome. This CRISPR cluster (positions 396,249–409,964) includes 47 repeats (5′ggtgcggttccttcgggagccgctcttcattgaggc3′), which are separated in groups of 3–20 repeats because of the insertion of five copies of IS256, one of which is actually integrated into an IS21 previously inserted in the cluster. Although none of the five putative Cas (CRISPR associated) proteins encoded upstream this CRISPR array are interrupted by any IS, the presence of an IS256 copy in the fifth CRISPR repeat in opposite transcriptional orientation supports that, at best, only the three spacers closer to the 5′ CRISPR could still be expressed and active against their corresponding (unknown) targets. Therefore, the following repeats and spacers of this CRISPR array would likely be non‐functional making them more susceptible of being invaded by ISs in the absence of counterselection pressure of viruses carrying the corresponding spacers. In contrast, the second CRISPR cluster (5,082,586–5,085,876, complementary strand) contains 45 repeats (5′gtgatctccgtccctgagacggagccgcattgcagc3′), preceded by five putative Cas proteins and does not contain any IS interrupting its continuity, supporting that this CRISPR‐Cas system is still active in defence against phages and/or plasmids (Nidhi et al., 2021) (Figure 1D).

### Presence of ESX systems in *M. hassiacum*

A main factor for the pathogenic character and HGT of many mycobacteria is the presence of Type Seven secretion systems (T7SS) known as ESX (Roy et al., 2020), involved in the secretion of small proteins containing the WXG motif. Five ESX systems (from ESX‐1 to ‐5) are encoded in *Mycobacterium tuberculosis*. In the genome of *M. hassiacum*, gene clusters encoding homologues to ESX‐1, ESX‐3 and ESX‐4 are present (see below), whereas no homologues of ESX‐2 and ESX‐5 were detected.

**FIGURE 2:**
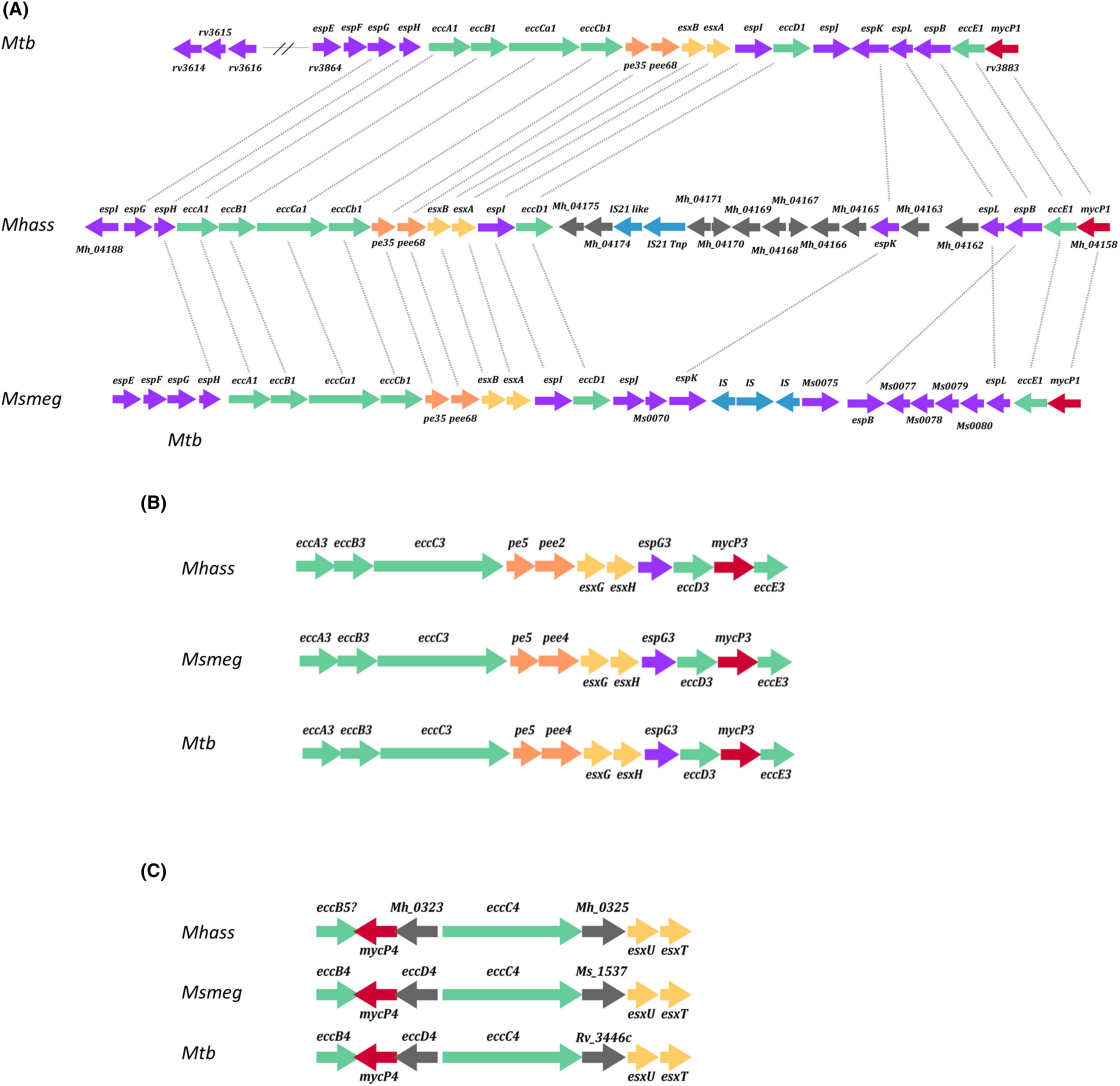
Analysis of EXC clusters of *Mycolicibacterium hassiacum*. (A) Figure shows a comparison between the genes coding for homologues to the ESX‐1 cluster in *M. hassiacum* (Mhass) and those found in *Mycobacterium tuberculosis* (Mtb) and *M. smegmatis* (Msmeg). Colour arrows indicate the position, direction and size of genes encoding homologues in the three strains. Homologues between the different strains are linked by dotted lines. (B) Figure shows a comparison between the genes coding for homologues to the ESX3 cluster in *M. hassiacum* (Mhass) and those found in *Mycobacterium tuberculosis* (Mtb) and *M. smegmatis* (Msmeg). Colour arrows indicate the position, direction and size of genes encoding homologues in the three strains. (C) Figure shows a comparison between the genes coding for homologues to the ESX‐4 cluster in *M. hassiacum* (Mhass) and those found in *Mycobacterium tuberculosis* (Mtb) and *M. smegmatis* (Msmeg). Colour arrows indicate the position, direction and size of genes encoding homologues in the three strains.

Protein factors secreted by ESX‐1 in *Mycobacterium tuberculosis* induce lysis of the phagosome membrane in macrophages and interact with different cytoplasmic proteins, subverting their normal protective activities, being thus required for its virulence (Wong, 2017). Actually, the BCG vaccine has a defective ESX‐1 secretion system, losing most of its pathogenicity (Nadolinskaia et al., 2020). The ESX‐1 gene cluster of *M. hassiacum* (Figure 2A) encodes homologues to some of these virulence‐related protein effectors like the EsxA/B porin‐forming proteins or the EspE and EspF proteins, whose actual roles in virulence in *Mycobacterium tuberculosis* are not well understood (Chirakos et al., 2020). However, as it happens in the non‐pathogenic *M. smegmatis*, the ESX‐1 gene cluster of *M. hassiacum* is interrupted by ISs and other genes, a trait that could contribute to the non‐pathogenic character of both strains.

By contrast, the gene cluster coding for homologues of ESX‐3 in *M. hassiacum* (Figure 2B) has a structure basically identical to those of *Mycobacterium tuberculosis* and *M. smegmatis*. In *Mycobacterium tuberculosis* this cluster is involved in metal homeostasis (Fe, Zn) through the secretion of proteins PE5 and PEE4 (Tufariello et al., 2016), which are also conserved in *M. hassiacum* and *M. smegmatis*. Actually, defective mutants of *M. smegmatis* in these genes require hemine for growth (Serafini et al., 2013), suggesting that this role could be likely conserved in *M. hassiacum*. Also, *Mycobacterium tuberculosis* proteins EsxG–EsxH, considered as virulence factors because they hinder the activity of Hrs ESCRT (endosomal sorting complex required for transport) factor, are present in *M. hassiacum* and *M. smegmatis*, supporting a pathogenesis—unrelated role for this cluster in the environment.

Finally, the *M. hassiacum* genome encodes a gene cluster with similar architecture to the ESX‐4 (Figure 2C), which has been shown to be involved in conjugation in different mycobacteria (Gray et al., 2016). In those strains in which the ESX‐4 system has been studied, it is induced in the recipient strain during conjugation through the extra‐cytoplasmic sigma factor SigM upon detection of conjugal contact in an ESX‐1 dependent manner (Clark et al., 2018), suggesting that *M. hassiacum* could function as recipient in the distributive conjugal transfer (DCT) mechanism characteristic of mycobacteria (Gray et al., 2016).

### The use of phytosterols by *M. hassiacum*

The thermophilic character of *M. hassiacum*, unique among mycobacteria, constitutes a putative major advantage for the biotransformation of highly valuable compounds of low solubility in water, such as sterols. Therefore, the ability of *M. hassiacum* to use sterols as carbon and energy source was assayed through growth‐dependent experiments in biotransformation medium containing soya phytosterols as the major carbon source (Experimental Procedures). After 5 days of growth, TLC analysis of the biotransformation medium revealed a clear decrease in the content of phytosterols, but without detecting any additional spots that could suggest the accumulation of steroidic intermediates (Supplementary Figure 3). These data demonstrated the actual use of phytosterols by *M. hassiacum* and suggested the existence of a biochemical pathway for its complete metabolization. Actually, we identified in the genome genes putatively involved in the metabolism of steroids by using the KEGG pathway 0984 as reference, with further refinement by BlastP comparisons and multiple sequence alignments. A total of 21 genes were identified as putative components of this pathway (Table 4). Among them, four ORFs encoded homologues of KstD, an enzyme described as 3‐keto steroid delta1‐dehydrogenase that could be a likely bottleneck in the pathway involved in the conversion of AD to ADD (https://www.genome.jp/pathway/map00984). In order to define which of these paralogs has more possibilities to codify the hypothesized enzyme, we carried out multiple sequence alignments of the paralogs with KstD proteins from different mycobacteria (Supplementary Figure 4). This lead us to select Mhass_03730 (584 amino acids, 1752 bp, locus 3,985,552–3,987,303) as the most likely KstD enzyme encoded in the genome of *M. hassiacum* (*kstD4*). We also obtained 3D models based on the X‐ray structure of PDB 4C3X, the KstD homologue from *Rhodococcus erythropolis* SQ1 (39.7% identity) and performed docking studies with the AD substrate since the lack of existing 3D models of those mycobacteria to use as the reference (Supplementary Figure 5A). In addition, we predicted the 3D model of *M. hassiacum* KstD4 with AlphaFold (https://alphafold.ebi.ac.uk/entry/A0A3P4A2I1). The resulting model, used as query, guides to the more recently reported 3‐keto steroid delta1‐dehydrogenase structure from *Sterolibacteriun denitrificans*, which included the ADD substrate (PDB 7P18) and share a 41.6% identity with KstD‐4 (Supplementary Figure 5B–D). These modelling studies support our selection of KstD4 as the most likely enzyme to convert AD to ADD in the pathway.

### Transformation of *M. hassiacum*

The analysis of HGT clues described above suggested that *M. hassiacum* could be susceptible of transformation with plasmids. However, it is known that most mycobacteria form clumps in liquid media, which generates difficulties in the preparation of electrocompetent cells (Campo‐Perez et al., 2021). To mitigate this effect, we grew *M. hassiacum* in GPHF and 7H10 agar media, and GPHF and 7H9 broths with or without OADC and with different amounts of Tween 80. Although it is possible to directly visualize the presence of clumps in the flasks, samples from various culture conditions were observed by bright field microscopy (*images not shown*) to identify those growth conditions in which the cells showed fewer and smaller aggregates. In all experiments with GPHF media the cells remained in aggregates, so we selected 7H9 medium with OADC and 0.15% Tween 80 as a suitable condition for growth without cell aggregation. These conditions were routinely used for the preparation of electrocompetent cells (Experimental Procedures).

To determine the proper antibiotic to be used for selection, cells grown under the optimized conditions were assayed on agar plates for their resistance levels to the thermostable antibiotics Str, Kan and Hyg at 55°C, a temperature at which the *M. hassiacum* grows efficiently. Under our selection conditions *M. hassiacum* did not show sensitivity to Hyg even at the highest concentration assayed (500 ng/μL), while complete inhibition of growth was observed for Kan and Str at 10 and 5 ng/μL, respectively. Similar results were obtained at 37°C but plates needed longer incubation times due to the low growth rates of *M. hassiacum* at this temperature (not shown). Thus, kanamycin and streptomycin were chosen for selection in our gene transformation assays.

Transformation trials were carried out with cells grown under the low aggregation conditions described above. DNA used for selection on Kan selection plates were replicative plasmid pJV53, or integrative plasmids pMC1s, and pTTP1B, whereas selection on Str plates was assayed after transformation with integrative plasmid pMY769 (Supplementary Table 2). The temperature for the incubation of the plates was set at 42°C as a compromise between increasing the growth rate of the strain and the thermosensitivity expected for antibiotic modifying and other plasmid‐encoded enzymes (Brouns et al., 2005). As shown in Table 3, the assays with replicative vector pJV53 and with integrative vector pMC1s did not result in Kan^R^ clones, thus being discarded. In contrast, transformation with integrative vector pTTP1B rendered a significant number of Kan^R^ colonies. The integration of this vector is likely to occur at positions with a unique sequence almost identical to the “Twetty” phage *att*B insertion site and located at positions 1,154,359–1,154,406, although we cannot disregard the putative presence of alternative integration sites for this vector in the genome in some of the clones. The best transformation efficiencies reached with the pTTP1B plasmid under these selection conditions (42°C) was around 10^4^ cfu/μg. As expected, when selection was carried out at 37°C, an increase in the transformation efficiency was observed (Supplementary Figure 6), a fact likely related to the thermosensitivity of its integrase and/or to that of the enzyme conferring Kan^R^, as commented above.

**TABLE 3:** Transformation assays on *M. hassiacum*. Assays were carried out with 300 ng of the indicated plasmids and selection plates were incubated at 42°C. Symbols indicate less than 50 colonies (+), between 50 and 200 (++), more than 200 (+++), or complete lawn (++++).

Plasmid	Antibiotic concentration (μg/ml)			
	0	12.5	20	30
pJV53 (replicative/Kan^R^)	++++	−	−	−
pMC1s (integrative L5, Kan^R^)	++++	−	−	−
pMY769 (integrative L5, Str^R^)	++++	++	++	+
pTTP1B (integrative Tweety, Kan^R^)	++++	+++	+++	+
no plasmid—Kan	++++	−	−	−
no plasmid—Str	++++	−	−	−

**TABLE 4:** Putative genes involved in steroids degradation in *M hassiacum.* Table shows the enzyme activity, its code in the *M. hassiacum* genome, the enzyme code number, the gene name and the position of the gene in the genome.

Enzyme name	Gene code	KEGG no	Enzyme	Size (aa)	Locus
Cholesterol oxidase	Mhass_00386	1.1.3.6	ChoD	575	420,539–422,266
3β‐hydroxysteroid dehydrogenase	Mhass_03213	1.1.1.145 (3β‐HSD)	—	362	3,401,387–3,402,475
Colest‐4‐en‐3‐one 26‐monooxygenase	Mhass_03703	1.14.15.28	—	404	3,927,003–3.928.217
Colest‐4‐en‐3‐one‐26‐monooxygenase	Mhass_01888	1.14.15.29	—	420	1,999,888–2,001,150
	Mhass_03637	1.14.15.29	—	409	3,864,055–3,865,284
	Mhass_03739	1.14.15.29	—	416	3,994,451–3,995,701
Steroid Δ isomerase	Mhass_03226	5.3.3.1	Ksi	123	3,424,752–3,425,123
	Mhass_03651	5.3.3.1	—	147	3,877,363–3,877,806
3‐ketosteroid delta 1‐dehydrogenase	Mhass_03730	1.3.99.4	KstD_4	583	3,885,552–3,987,303
3‐keto‐5α‐steroid‐4‐dehydrogenase	Mhass_02476	1.3.99.5	—	528	2,621,394–2,622,980
3‐ketosteroid 9 α‐monooxygenase	Mhass_02479	1.14.15.30	KshA_4	385	2,624,124–2,625,281
	Mhass_03697	1.14.15.30	KshA_8	379	3,920,249–3,921,388
	Mhass_03711	1.14.15.30	KshA_9	384	3,936,408–3,937,562
	Mhass_03780	1.14.15.30	Hmp	353	4,036,126–4,030,187
3‐hydroxy‐9,10‐secoandrosta‐1,3,5(10)‐triene‐9,17‐dione monooxygenase	Mhass_03776	1.14.14.12	HsaB	189	4,032,326–4,032,895
	Mhass_03779	1.14.14.12	HsaA3	394	4,034,724–4,035,908
3,4‐dihydroxy‐9,10‐secoandrosta‐1,3,5(10)‐triene‐9,17‐dione 4,5‐dioxygenase	Mhass_03777	1.13.11.25	HsaC_2	302	4,032,892–4033,800
4,5:9,10‐diseco‐3‐hydroxy‐	Mhass_03778	3.7.1.17	HsaD_5	304	4033.797
‐5,9,17‐trioxoandrosta‐1(10),2‐diene‐4‐oate hydrolase					4,034,711
3‐[(3aS,4S,7aS)‐7a‐methyl‐1,5‐	Mhass_01072	6.2.1.41	FadD3_4	485	1,131,688
‐dioxo‐octahydro‐1H‐inden‐4‐il]					1,133,145
propanoate‐CoA ligase	Mhass_03132		—	497	3,322,992–3,324,485
	Mhass_03765		FadD3_6	513	4,019,741–4,021,282

Transformation with the pMY769 integrative vector also allowed the selection of Str^R^ colonies (Table 3) but with lower efficiencies compared to pTTB1B. This could be related either with the limited sequence identity of the two putative insertion sites homologous to the phage “L5” *attB* site used by this vector (positions 1,265,108–1,265,150 and 1,214,963–1,215,005), and/or to the lower thermostability of the enzyme conferring Str resistance encoded by the plasmid. Nevertheless, our data support the possible use of Str as a second selection marker for *M. hassiacum*.

In addition to electroporation, we also attempted to generate a transposon library of random mutants using the PhiMycoMartT7 phage as previously described for other species of the genus (Bardarov et al., 2001; Siegrist & Rubin, 2009). However, we did not obtain any Kan^R^ clones after infection, in contrast to the presence of a large number of colonies in the *M. smegmatis* control strain. Moreover, infection assays at lower multiplicity also failed to generate plates in *M. hassiacum*, so we checked by electron microscopy if the phage was able to bind to the bacteria. As shown in Supplementary Figure 7 we did not detect any bona fide interaction between the phage and *M. hassiacum* cells, suggesting the lack of adsorption of the phage to the surface.

### Isolation of kstD4 mutants

As the optimization of our transformation procedure was carried out using antibiotic (Kan)‐modifying genes from mesophiles, and the comparisons between efficiencies of selection on Kan plates at 37 versus 42°C suggested limitations in the thermostability of the antibiotic selection markers, we leveraged an existing thermostable resistance marker suitable for use at 55°C, the optimum temperature for growth of *M. hassiacum*. Therefore, we constructed vector pMH3, which is selectable in *E. coli* by resistance to ampicillin at 37°C, and it also includes a gene cassette encoding a thermostable variant of a kanamycin nucleotidyl transferase that works well up to 70°C (Lasa et al., 1992). To ensure enough expression of this resistance enzyme from a single copy in the chromosome of *M. hassiacum* we used the promoter that directs the expression of the mesophilic kanamycin resistance (selectable up to 42°C) in plasmid pTTP1B.

**FIGURE 3:**
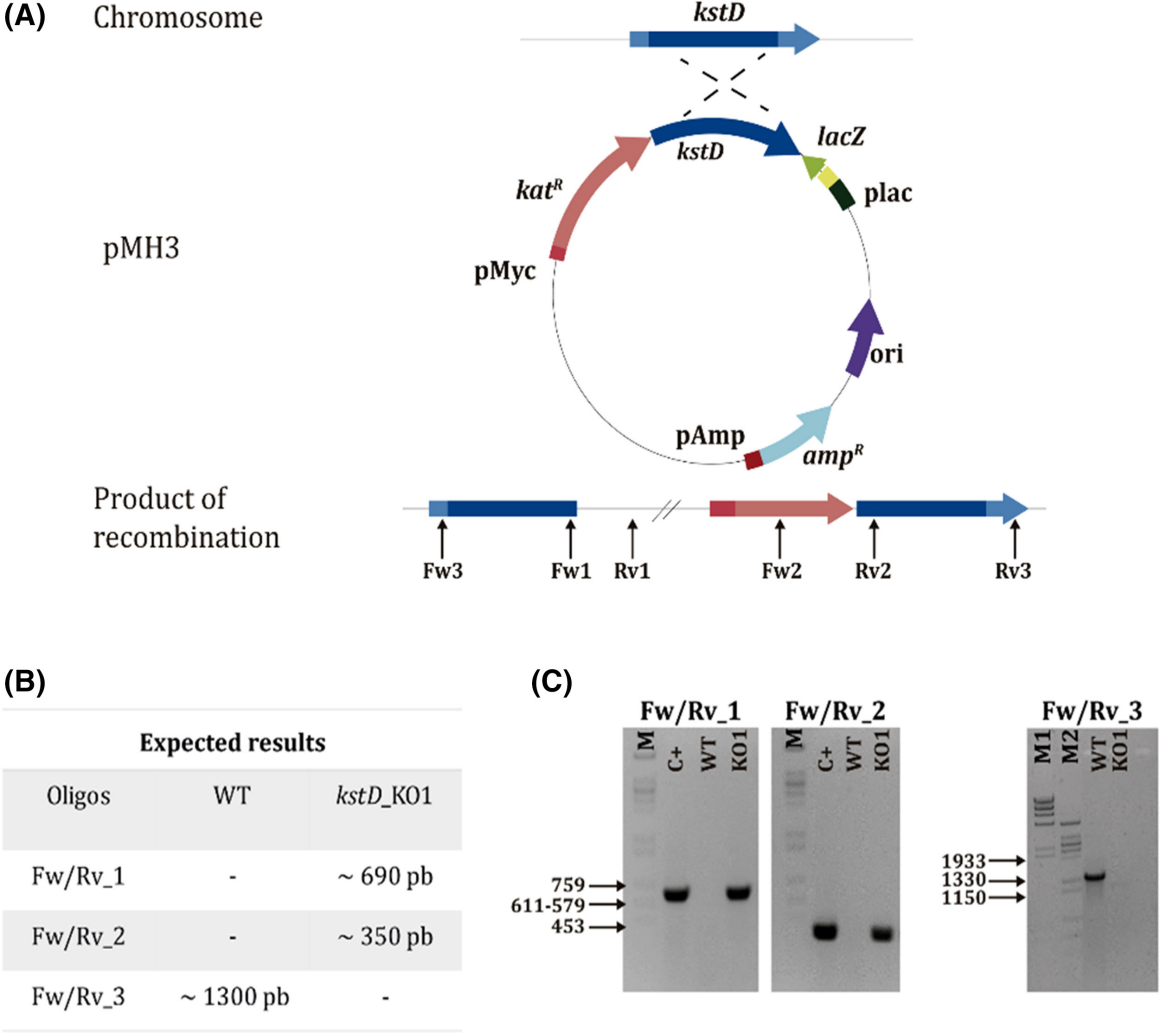
Isolation of the *kstD*_KO1 mutant. (A) Scheme of the recombination and annealing position of the primers used to confirm the mutation. (B) Expected size of the amplification products for the WT and *kstD*_KO1 mutant with primer pairs kstD_KO_Fw/Rv1, kstD_KO_Fw/Rv2 and kstD_KO_Fw/Rv3. (C) Agarose gel electrophoresis showing the expected size for the different amplicons obtained for WT and KO1 mutant.

The isolation of a knockout mutant lacking the putative KstD4 activity (*kstD*_KO1) was carried out through a single crossover insertion strategy by using an internal fragment of 1260 bp included in the integrative vector pMH3::*kstD4* as substrate for homologous recombination (Experimental Procedures). Upon transformation, only two colonies were recovered from 7H10 plates with Kan. Both were grown in 7H9 liquid medium with Kan, and the genomic DNA was extracted and analysed by PCR to confirm the mutation. As shown in Figure 3, amplification with primer pairs kstD_KO_Fw/Rv1 and kstD_KO_Fw/Rv2 (Supplementary Table 2) produced the expected products of 690 and 350 bp from the genome in mutant KO1, whereas no amplification was detected when the genome of the wild type parental was used as template. On the other hand, amplification with primers kstD_KO_Fw/Rv3 produced the expected 1300 bp amplicon in the wild type, and no amplification in the mutant, where the distance between the primer hybridization sites was too large for amplification. Results from the second putative mutant colony discarded integration of the suicide plasmid at the expected site and was discarded for further analysis, so only mutant *kstD*_KO1 was used for further analysis.

### Phytosterols degradation by the kstD_KO1 mutant

**FIGURE 4:**
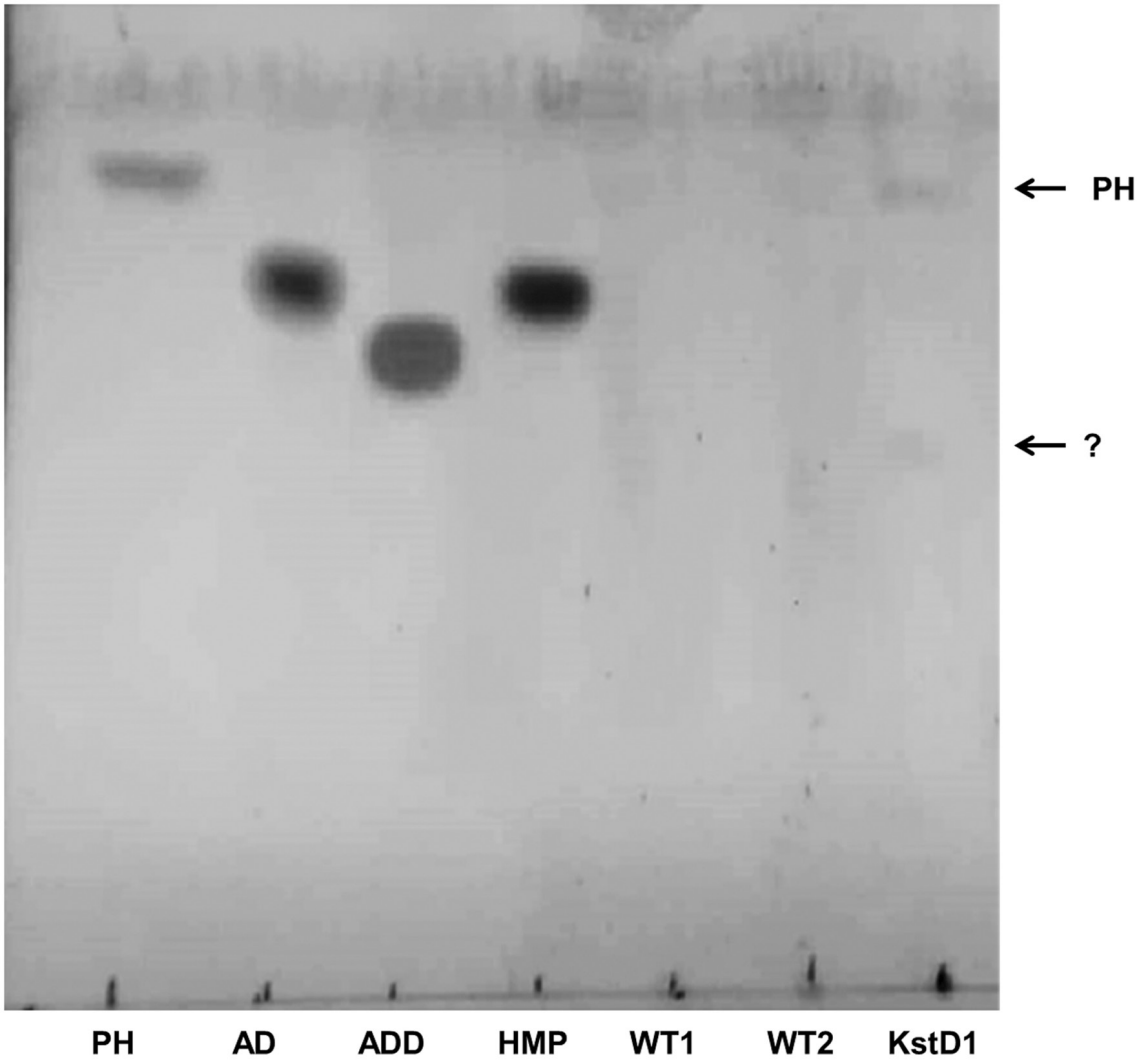
Analysis and comparison of phytosterols content in bioconversion media by TLC for WT and *kstD*_KO1 mutant. Lines correspond to: PH, phytosterols standard; AD, AD standard; ADD, ADD standard; HPM, 20‐hydroxymethylpregn‐4‐ene‐3‐one (20‐HMP) standard; WT1 and WT2, duplicates of WT biotransformation media; KstD1, *kstD*_KO1 biotransformation media. Each line contains 10 μl of extract. For standards, 10 μg were disposed of in each case. Extracts from samples were equivalent to an initial phytosterols content of 1 μg. The question mark indicates the potential intermediates whose presence is not detectable in WT lines.

**FIGURE 5:**
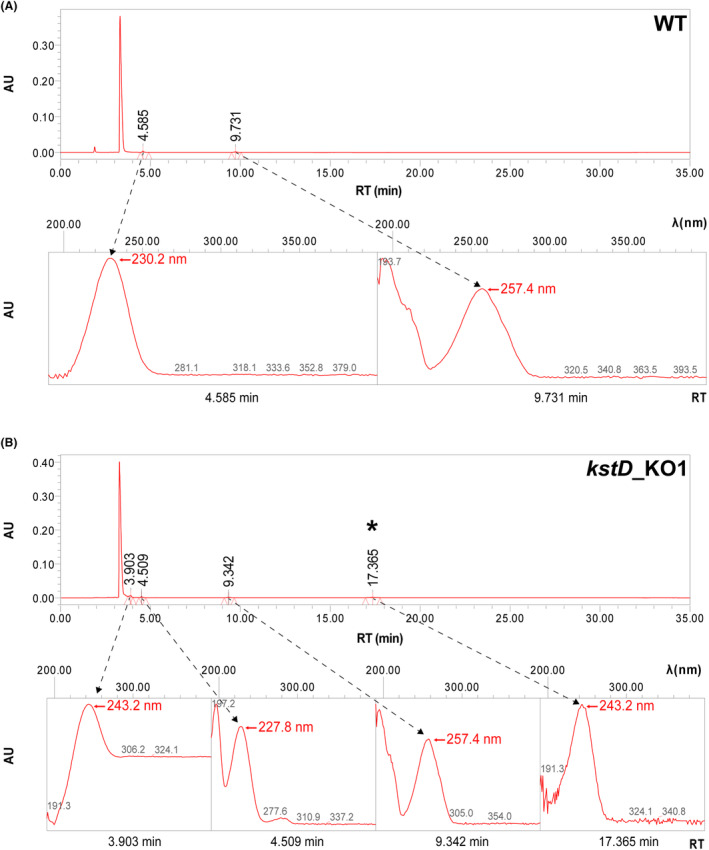
HPLC analysis of biotransformation media. Chromatogram and absorption spectra of detected peaks from (A) extract from wild type (WT) and (B) extract from mutant (*kstD*_KO1). Each chromatogram (top plots) shows the detected peaks, and their retention time (RT) in minutes. Bottom plots represent the adsorption spectrum of each peak, identified by a dashed arrows, in the wavelength typical for steroid molecules (bottom plots). The asterisk indicates the steroid compound present in the mutant bioconversion media and absent in the WT.

Metabolic blocks preventing activity of KstD may provide effective accumulation of useful intermediates such as AD or ADD (Bragin et al., 2019; Tekucheva et al., 2022). To check this possibility, growing assays in the biotransformation medium were performed with the *kstD*_KO1 mutant in comparison with the wild type. Despite its low sensitivity, TLC analysis of the medium showed different sterol profiles between the wild‐type strain and the *kstD*_KO1 mutant after 5 days of growth (Figure 4). First, almost no phytosterol spot was detected in the wild‐type, whereas in the mutant a faint spot was still detected (Figure 4), suggesting a limitation in phytosterols degradation. In addition, a compound with faster mobility than androstenedione (AD) was detected in the *kstD*_KO1 mutant and not in the wild type, suggesting the accumulation of a degradation product. To check its nature, we further analysed the medium by HPLC (Figure 5). In comparison with the profile obtained in the wild type, two additional products were detected in the chromatogram of *kstD*_KO1 mutant whose respective retention times (RT) did not match either with AD or ADD. However, one of these products (retention time of 17.36 min) exhibited significant absorbance at 243 nm, typical of steroid compounds.

**FIGURE 6:**
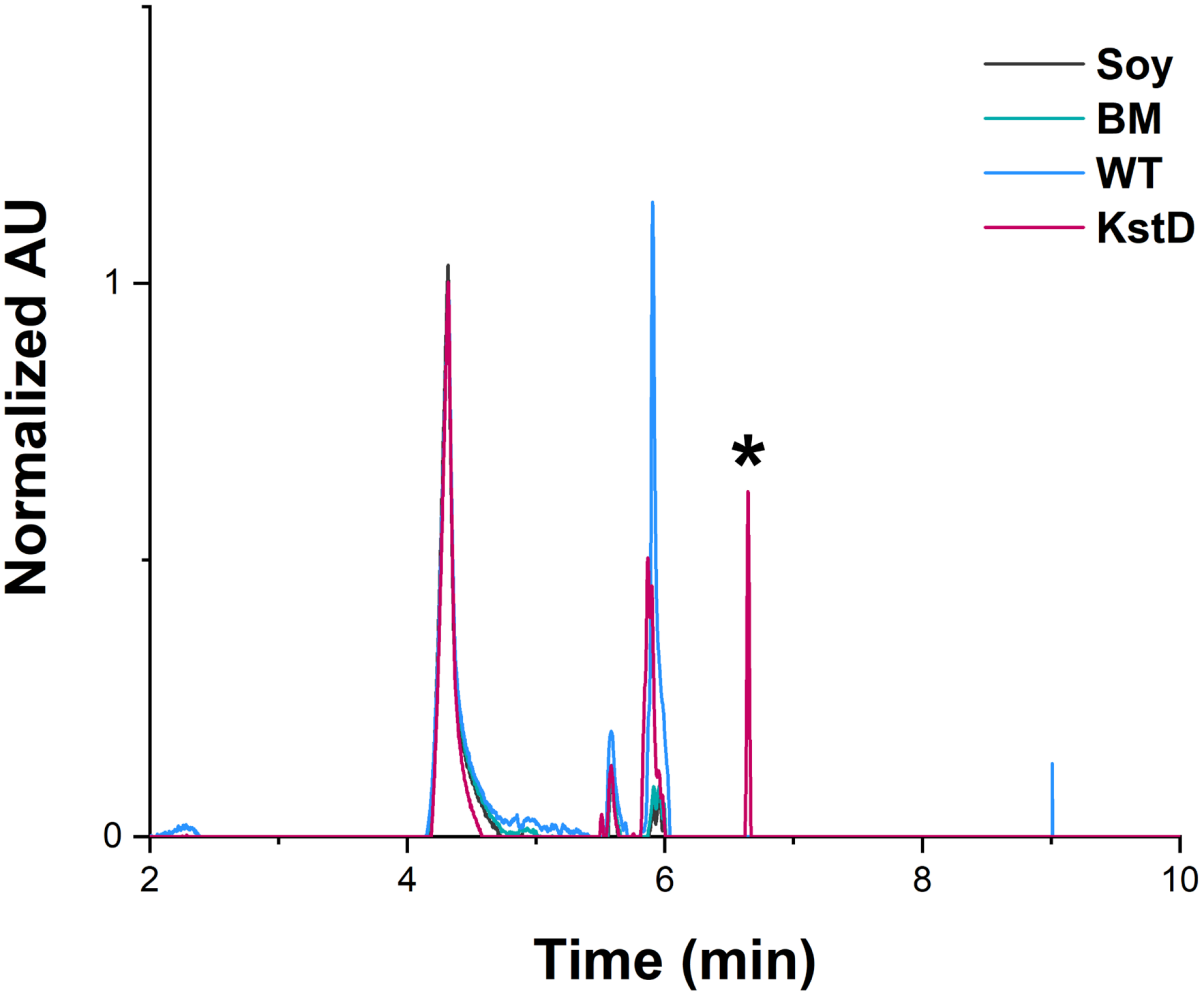
Representation of stacked chromatograms from UPLC‐MS analysis. Absorption spectra at 243 nm. Values are normalized to the one obtained at an RT of 4.32 min. For better visualization of the results the figure illustrates peaks eluted from 2 min. Soy; soy standard, BM; control biotransformation media without *Mycolicibacterium hassiacum*, WT; biotransformation media from wild type *M. hassiacum* and KstD; biotransformation media from the *M. hassiacum* mutant *kstD*_KO1. The asterisk highlights the potential intermediate accumulated by the mutant in the BM.

In order to elucidate the mass of the compounds accumulated in the *kstD*_KO1 mutant, samples of larger volume biotransformation assays were also analysed by UPLC‐MS. The evaluation of chromatograms showed different chromatographic profiles of the biotransformation media of WT and *kstD*_KO1 mutant at around 6 min of RT (Supplementary Figure 8A), which confirms a different metabolism of the starting substrate. A major product of a retention time (RT) of 6.35 min appeared in the *kstD*_KO1 mutant that was not accumulated in the wild type (Figure 6). Although the specific mass of this compound could not be clearly identified due to excessive background in the mass spectra (Supplementary Figure 8C), its RT was similar to that of the common intermediates AD and ADD (6.56 and 6.33 min, respectively, Supplementary Figure 8B) suggesting that a molecule of similar polarity is being produced.

## CONCLUSIONS

The thermophilic character of *M. hassiacum* could facilitate its biotechnological use with water insoluble and low‐cost substrates such as phytosterols to obtain steroid pharmaceuticals as shown with mesophilic mycobacteria (Zhao et al., 2021). Also, higher thermal stability in proteins is associated with easier generation of crystals for structural analysis and facilitate further in silico applications (Cava et al., 2009) in search for specific inhibitors that could have antibiotic effects against pathogenic mycobacteria. However, the first step toward the use of *M. hassiacum* as a laboratory and industrial model needs for an accurate genomic characterization and the development of gene manipulation protocols, as described for the first time in this work.

Although the high error rate for the single molecule real‐time sequencing (SMRT) sequencing of PacBio Biosciences was already known, the presence of systematic errors at specific points was not described until recently (Sacristán‐Horcajada et al., 2021). Our analysis revealed that these errors were associated to specific G/C tracks without a defined short range sequence environment. Because of the relevance of this systematic bias, our results show that the combination of long reads and massive short reads technologies should be a required gold standard before deposition in the gene banks, at least for high GC content genomes (Sacristán‐Horcajada et al., 2021).

Absence of plasmids has been assumed to be a common trait among mycobacteria. However, recent bioinformatics analysis of 242 partial and complete genomes belonging to 69 defined species and undefined species isolates of the *Mycolicibacterium* genus revealed the presence of plasmids in more than 50% of them (Morgado & Vicente, 2021). Although this study did not include any *M. hassiacum* isolate, our results support that the strain analysed in this work does not have any large plasmid. In agreement with this, T7SS secretion systems ESX‐2 and ESX‐5, usually associated to the presence of large plasmids in the *Mycolicibacterium* genus (Morgado & Vicente, 2021), were not identified in *M. hassiacum*. Also, as all the short Illumina sequences could be paired to the PacBio assembly, the putative presence of small plasmids was also disregarded.

Despite the absence of plasmids, the analysis of the *M. hassiacum* genome suggests the existence of frequent events of horizontal gene transfer. On the one hand, the presence of two CRISPR arrays supports the existence of events of infections by mycophages, and on the other hand, the high number and diversity of ISs, some of them present in other genera, supports the ability of the strain to acquire genes from other microorganisms. Our data also support that at least some of these ISs are active, for example those interrupting the CRISPR1 array. Finally, the presence of ESX‐1 and ESX‐3 type‐7 secretion systems, involved in Distributive Conjugal Transfer in mycobacteria (Gray et al., 2016), are present in the *M. hassiacum* genome, it is tempting to speculate that this mechanism of HGT is active in *M. hassiacum* and putatively responsible for the acquisition of ISs from other mycobacteria.

To manipulate *M. hassiacum* we first demonstrated its transformability by using thermostable antibiotics Str and Kan for selection at 55°C. However, as the plasmids used for transformation were derived from mesophilic mycobacteria, we started our transformation assays by selecting at 42°C with both replicative and integration‐specific suicide plasmids, as a compromise between expected maximum thermostability of the selection genes encoded by the plasmids and enough growth rate of the strain. Once the conditions to reach appropriate transformation efficiencies were reached, specially associated to integrative vector pTTP1B, we replaced the thermosensitive Kan phosphotransferase by a thermostable version of a Kan nucleotidyl transferase which is active at much higher temperatures (Lasa et al., 1992) and used it for the selection of targeted knockout genes at 55°C.

The ability of *M. hassiacum* to use phytosterols and the availability of a curated genome allowed us to propose a putative degradation pathway that included 21 putative genes. Many of these hypothetical genes were predicted to encode similar activities. For example, we identified genes encoding up to three putative cholest‐4‐en‐3‐one 26‐monooxygenases, two putative sterol delta‐isomerases, four putative 3‐ketosteroid 9 α‐mono‐oxygenases and two putative 3‐hydroxi‐9,10‐secoandrosta‐1,3,5(10)‐triene‐9,17‐dione monooxygenases, advising for the need of bioinformatics, biochemical and genetic analysis to decipher the differences in the activities between these hypothetical isoenzymes. To ensure that the attempted gene knockout was likely to result in a detectable phenotype, we focussed on the Mhass_03730 gene, which encodes the most likely KstD homologue (KstD4) present in *M. hassiacum* and that could constitute a metabolic bottleneck in our predicted pathway: the conversion of AD to ADD. We got the expected mutant by single recombination using a suicide vector with the thermostable marker developed in this work. Such kind of single crossover mutations do have polar effects on downstream genes, so future developments of thermostable versions of counter selectable markers similar to that used for *M. tuberculosis* and *M. smegmatis* (Barkan et al., 2011) could constitute a major step towards the use of this organism as a laboratory and industrial model.

Despite these limitations, the *kstD*_KO1 mutant obtained was much less efficient than the wild type strain in the degradation of phytosterols, as hypothesized by our biodegradation model. However, our UPLC‐MS data did not allow us to define the nature of the major sterol intermediates accumulated in the mutant that despite similar retention times was not either AD or ADD, precluding a clear identification of the role of *kstD4* gene, but did probe that genetic manipulation of this thermophilic mycobacterium is now feasible.

## AUTHOR CONTRIBUTIONS

**Mercedes Sánchez‐Costa:** Data curation (equal); investigation (equal); methodology (lead); writing – original draft (equal). **Susanne Gola:** Data curation (supporting); methodology (supporting); writing – review and editing (supporting). **Marta Rodríguez‐Sáiz:** Methodology (supporting); writing – review and editing (supporting). **José‐Luis Barredo:** Methodology (supporting); resources (supporting); writing – review and editing (supporting). **Aurelio Hidalgo:** Conceptualization (equal); funding acquisition (supporting); writing – review and editing (equal). **José Berenguer:** Conceptualization (equal); funding acquisition (lead); project administration (lead); writing – original draft (lead); writing – review and editing (lead).

## FUNDING INFORMATION

This research was funded by the Spanish Ministry of Science and Innovation, grant number PID2019‐109073RB‐I00 and the European Union project GA 685474‐2.

## CONFLICT OF INTEREST STATEMENT

Authors declare that they have no conflict of interest in this research.

## Supporting information


Supporting information S1
