# Supplementary material for: From accurate genome sequence to biotechnological application: The thermophile Mycolicibacterium hassiacum as experimental model
Source: Microb Biotechnol. 2023 Jul 27;17(1):e14290. doi: 10.1111/1751-7915.14290 (PMC10832570; doi:10.1111/1751-7915.14290)
Supplement: Supplementary file 1 — Supporting information S1 [file MBT2-17-e14290-s001.docx]

**Revised version 2 to MICROBIO-2022-218-RA**

SUPLEMENTARY MATERIAL TO:

***From accurate genome sequence to biotechnological application: the thermophile Mycolicibacterium hassiacum as experimental model.***

Mercedes Sánchez-Costa^1^, Susanne Gola^2^, Marta Rodríguez-Sáiz^3^, José-Luis Barredo^3^, Aurelio Hidalgo^1^, and José Berenguer*^1^

Contents:

Supplementary Figure 1

Supplementary Figure 2

Supplementary Figure 3

Supplementary Figure 4

Supplementary Figure 5

Supplementary Figure 6

Supplementary Figure 7

Supplementary Figure 8

Supplementary Table 1

Supplementary Table 2

**Supplementary Figure 1.** Motifs found through the comparison of 20 bases upstream and downstream of the sites misread by PacBio. Logo size represents relative e-value against position as revealed by MEME.

**Supplementary Figure 2.** KEGG pathways classification that includes more than 10 ORFS of *M. hassiacum* genome annotation. The X axis indicates the KEGG classification for each pathway, against the number of genes assigned for each of them in the Y axis.


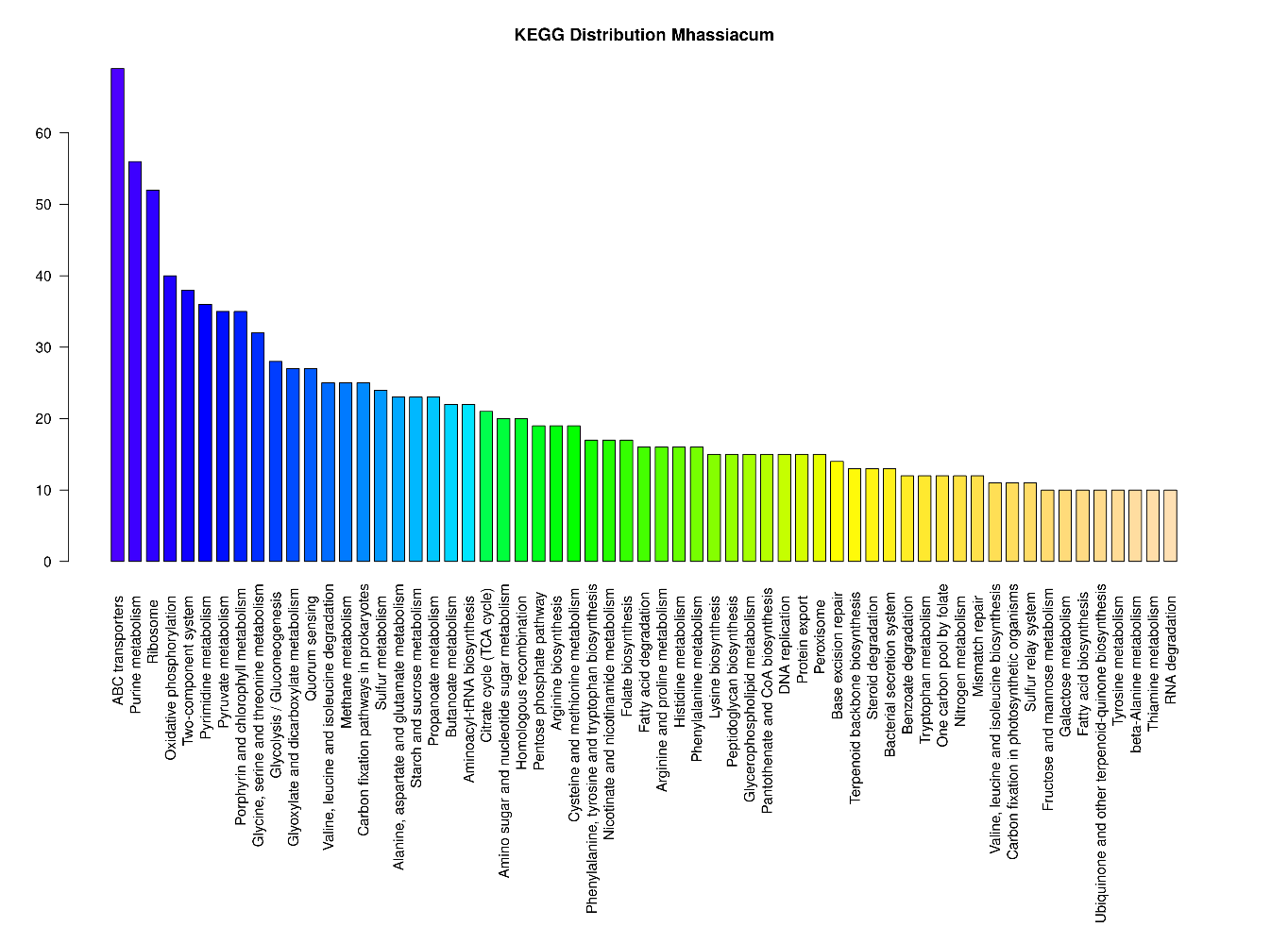


**Supplementary Figure 3.** Analysis of phytosterols as carbon source in biotransformation media by TLC. Line 1, AD standard (0.1 mg); line 2, phytosterols standard (0.01 mg, main component in the extraction β-sitosterol); lines 3 and 4, duplicates of the extraction of the BM corresponding to the control without *M. hassiacum* (0.02 mg), and lines 5 and 6, duplicates of the extraction of the biotransformation media. Samples for the TLC were extracted after five days of incubation at 55 °C.

**Supplementary Figure 4.** Amino acid sequence alignment of 3-Ketosteroid delta1-dehydrogenases. KstD of *Rhodococcus. erythropolis* SQ1 (4C3X_A), KstD of *M. smegmatis* (MSMEG_5941), KstD of *ycobacterium tuberculosis* (Rv3537), KstD of *Sterolibacterium denitrificans* (7P18) and the proteins of *M. hassiacum,* KstD1 (Mhass_0710), KstD2 (Mhass_2480), KstD3 (Mhass_2630) and KstD4 (Mhass_3730).


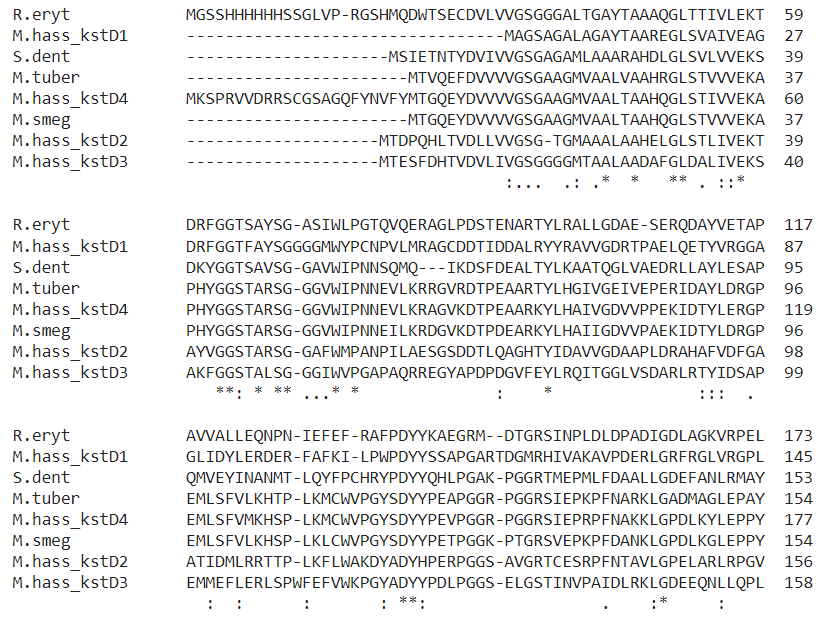

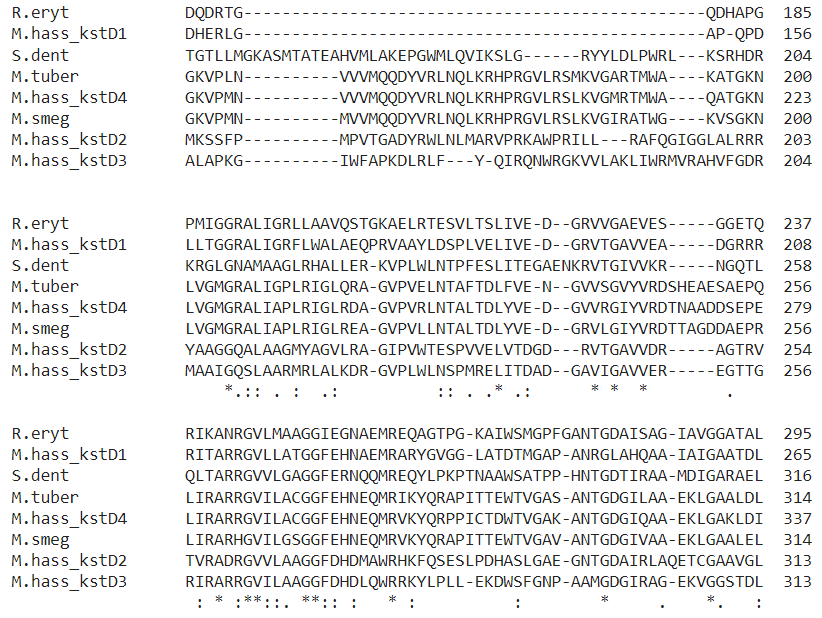

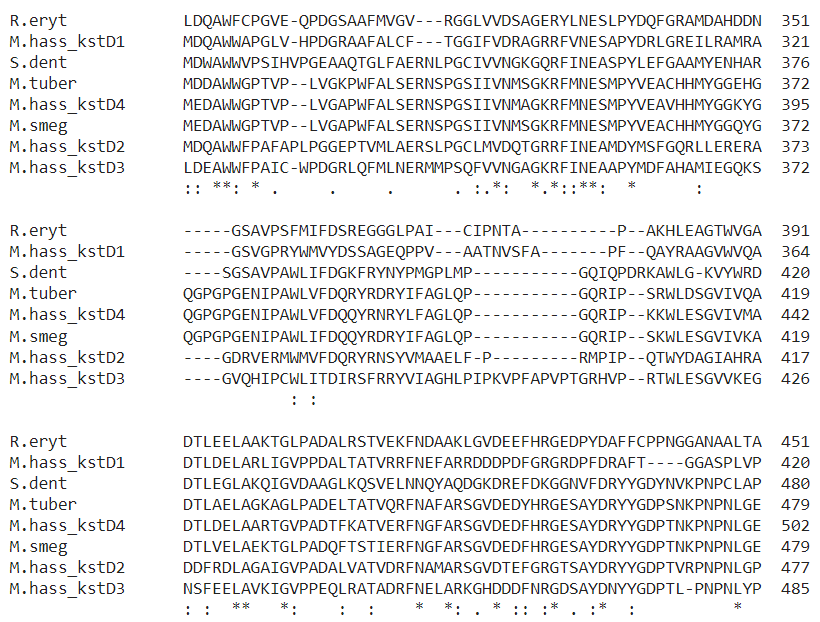

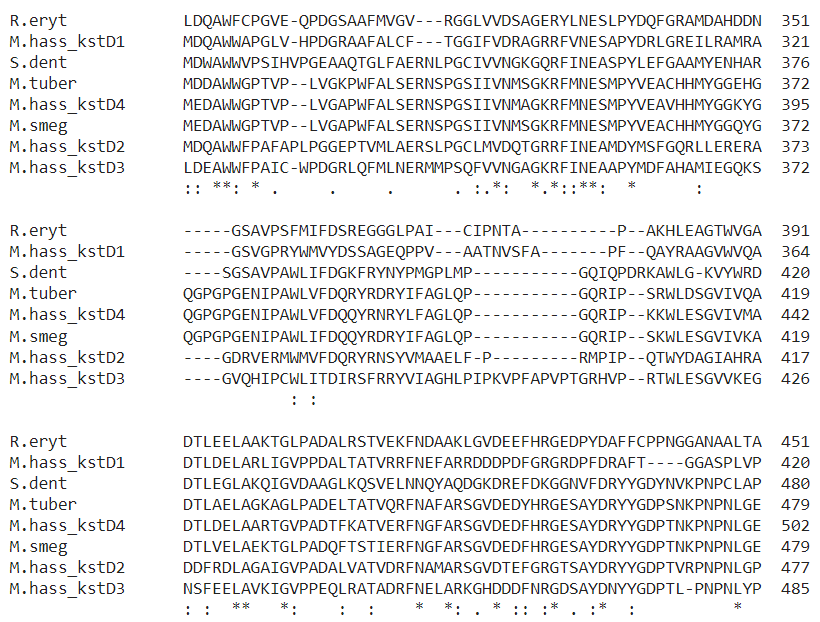


#### Supplementary Figure 5. 3D models of a putative protein of M. hassiacum homologue to KstD. A) Model of the protein encode by gene of code Mhass_03730 using as reference the 3-ketosteroid delta1-dehydrogenase from R hodococcus erythropolis SQ1 (PDB 4C3X, 39.72 % identity). The model is presented with the AD substrate and FAD cofactor. B) Overlayed 3D models of the protein Mhass_03730 predicted by AlphaFold (Blue) and the reference KstD from Sterolibacterium denitrificans (7P18). C) Individual structure of KstD from S. denitrificans (7P18) and D) individual model of Mhass_03730 predicted by AlphaFold. Models from B-D are presented with the ADD substrate and FAD cofactor.


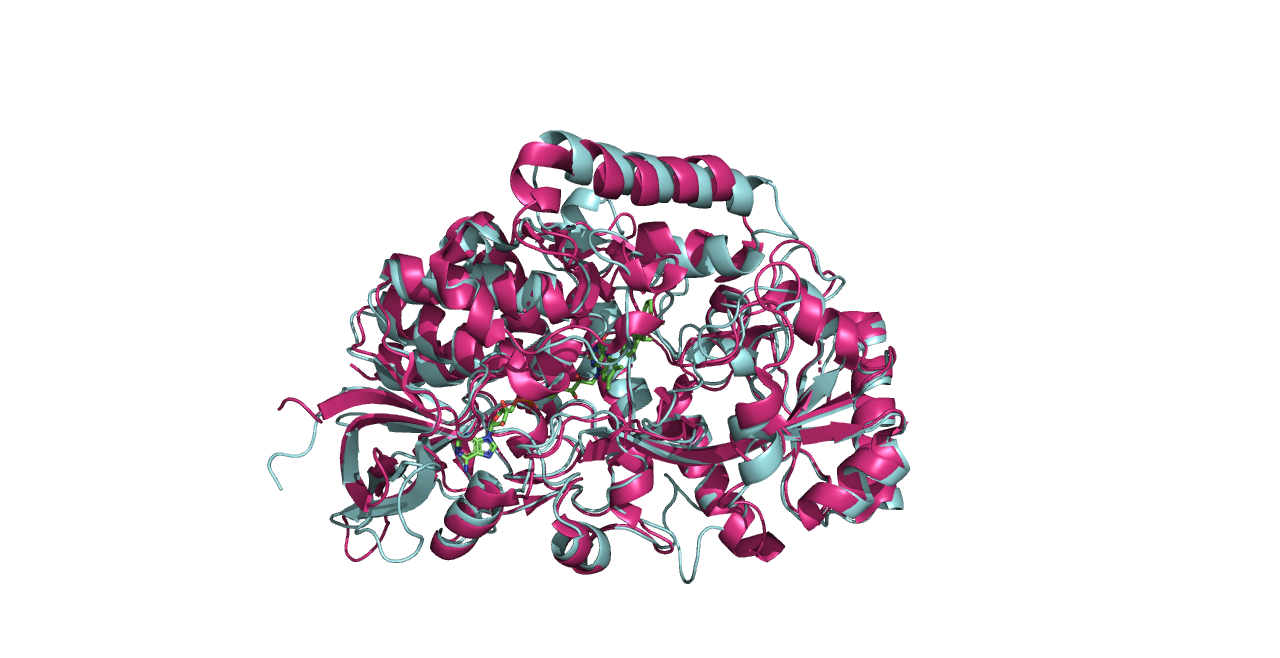

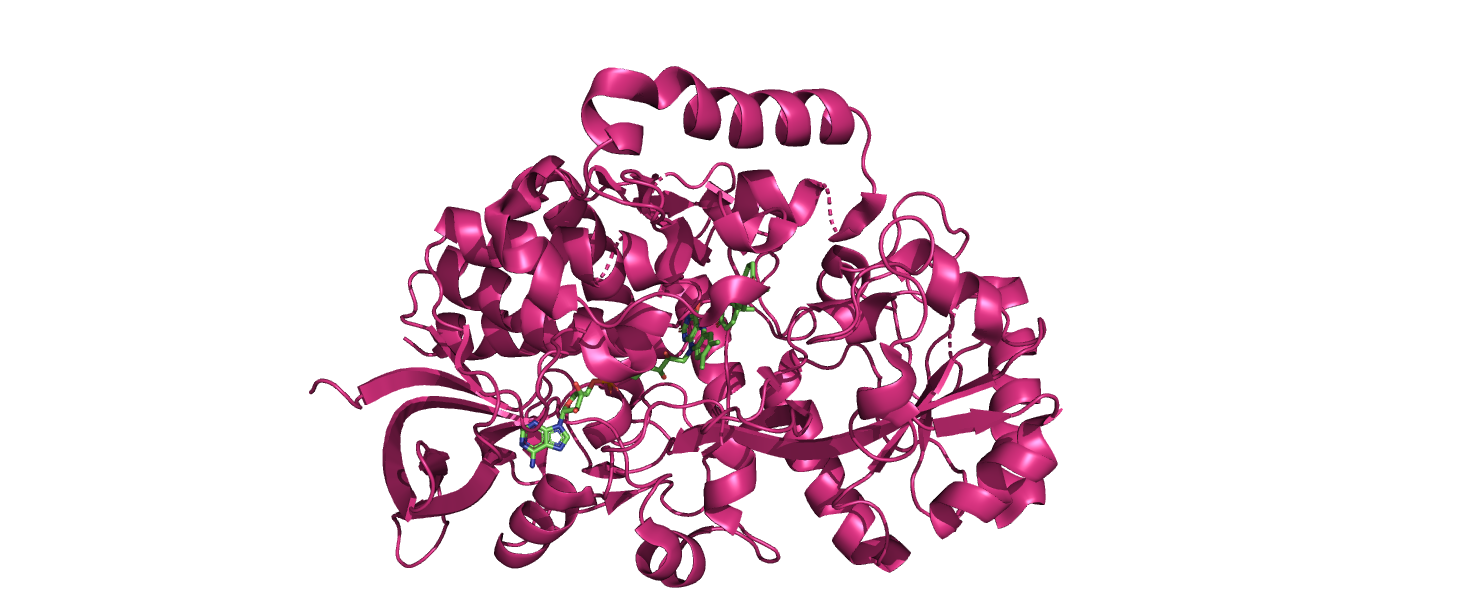

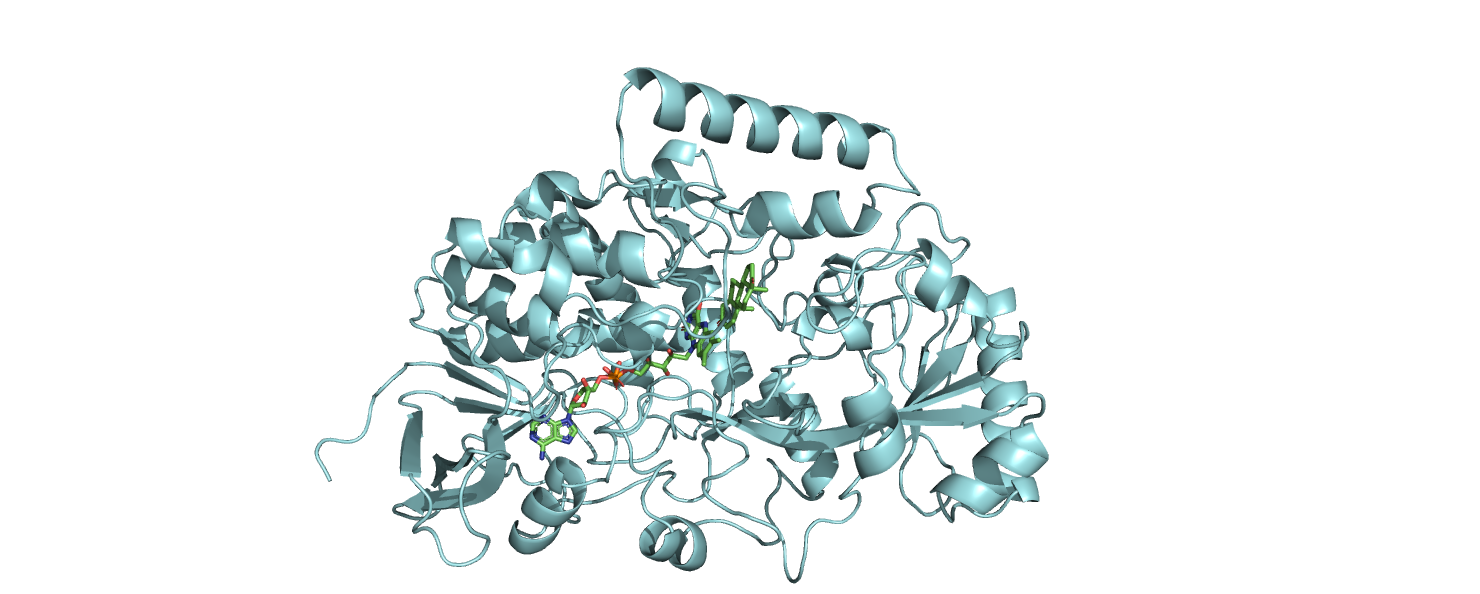


**A.**

**B.**

**C.**


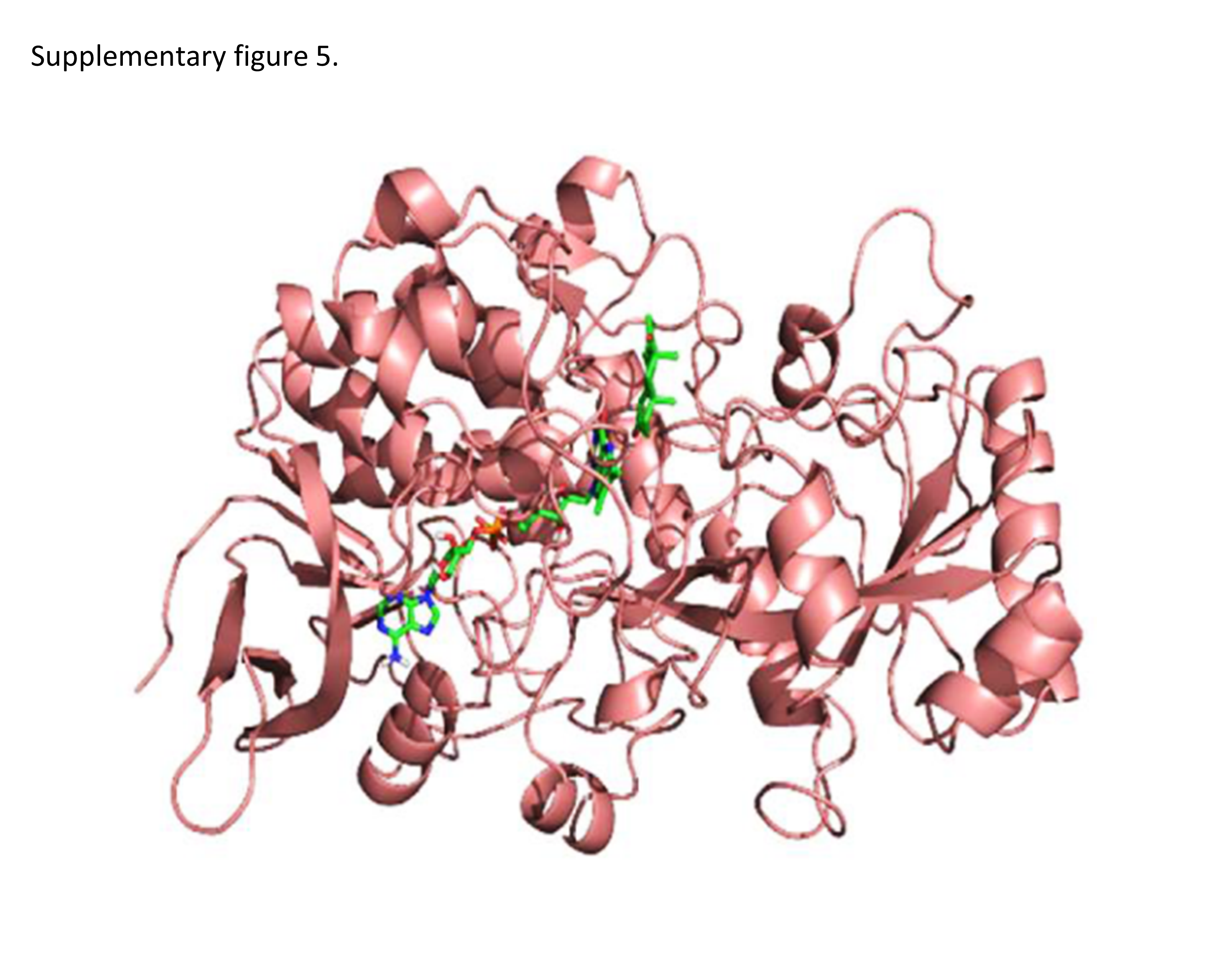


**D.**

**Supplementary Figure 6.** Effect of selection temperature on transformation efficency *of M. hassiacum.* Images of selection plates with kanamycin incubated with *M. hassiacum* transformed by electroporation with pTTP1B incubated at 42 °C (left) or at 37 °C (right ) for 7 days.

**Supplementary Figure 7.** Electron microscopy images of PhiMycoMarT7 and *M. hassiacum*. 1) 20,000x magnification, scale 100 nm, 2) 50,000x magnification, scale 90 nm and 3) 80,000x magnification, scale 60 nm. Cells observed after infection.

#### Supplementary Figure 8. UPLC raw results. A) Chromatograms at absorbance 243 nm of soy standard, biotransformation media without bacteria, biotransformation media from wild type M. hassiacum and biotransformation media from the M. hassiacum mutant kstD_KO1. B) Chromatograms at absorbance 243 nm of common intermediates AD and ADD. C) Mass spectrum of the compound eluted at 6.35 min from the kstD_KO1 bioconversion media.


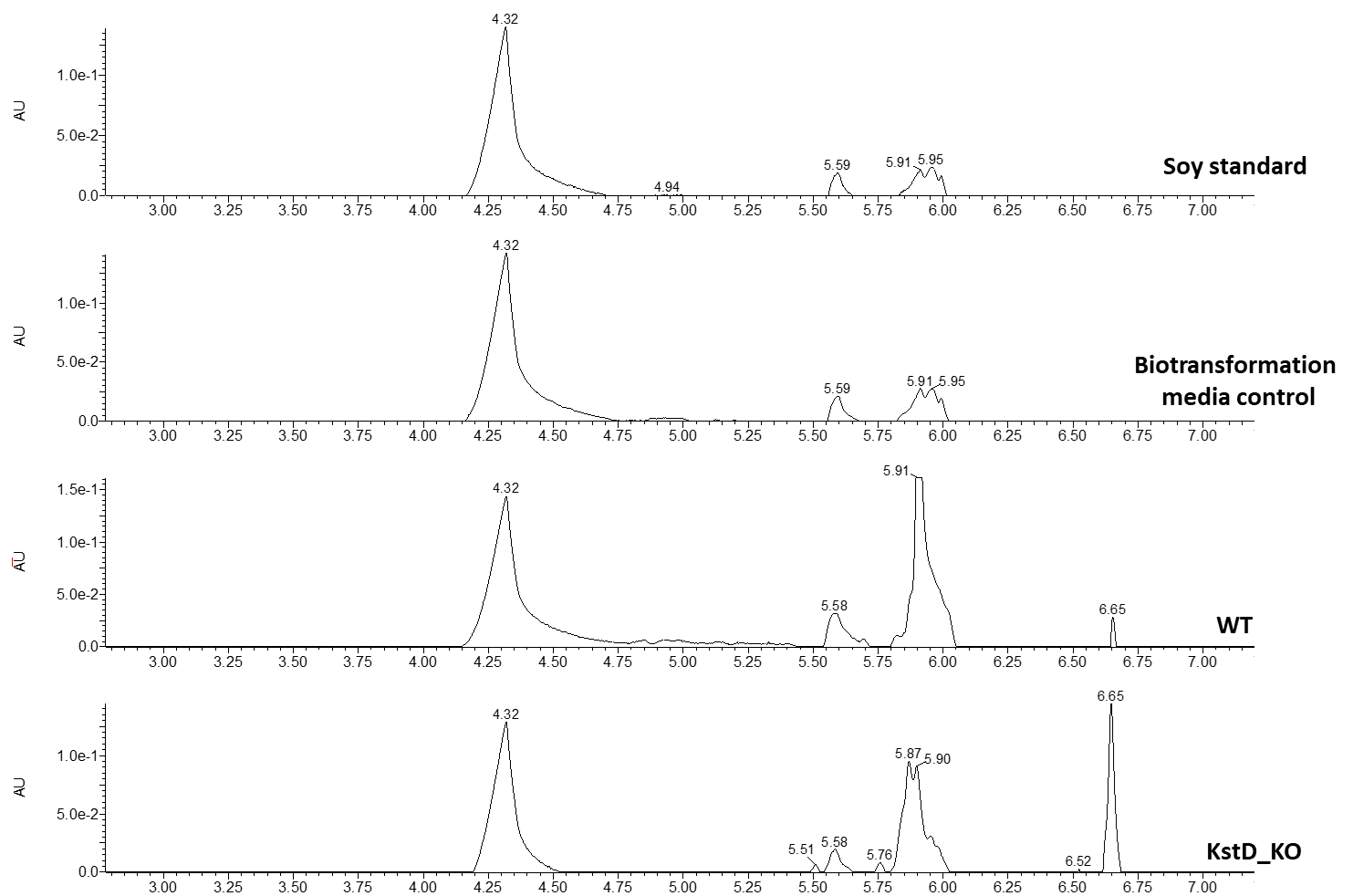


**A.**

**C.**


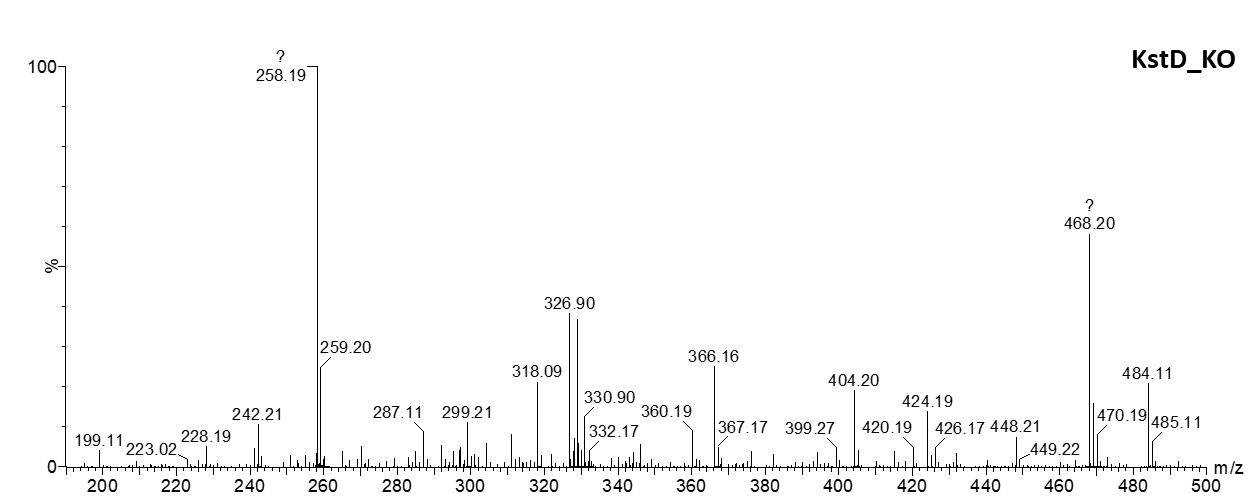

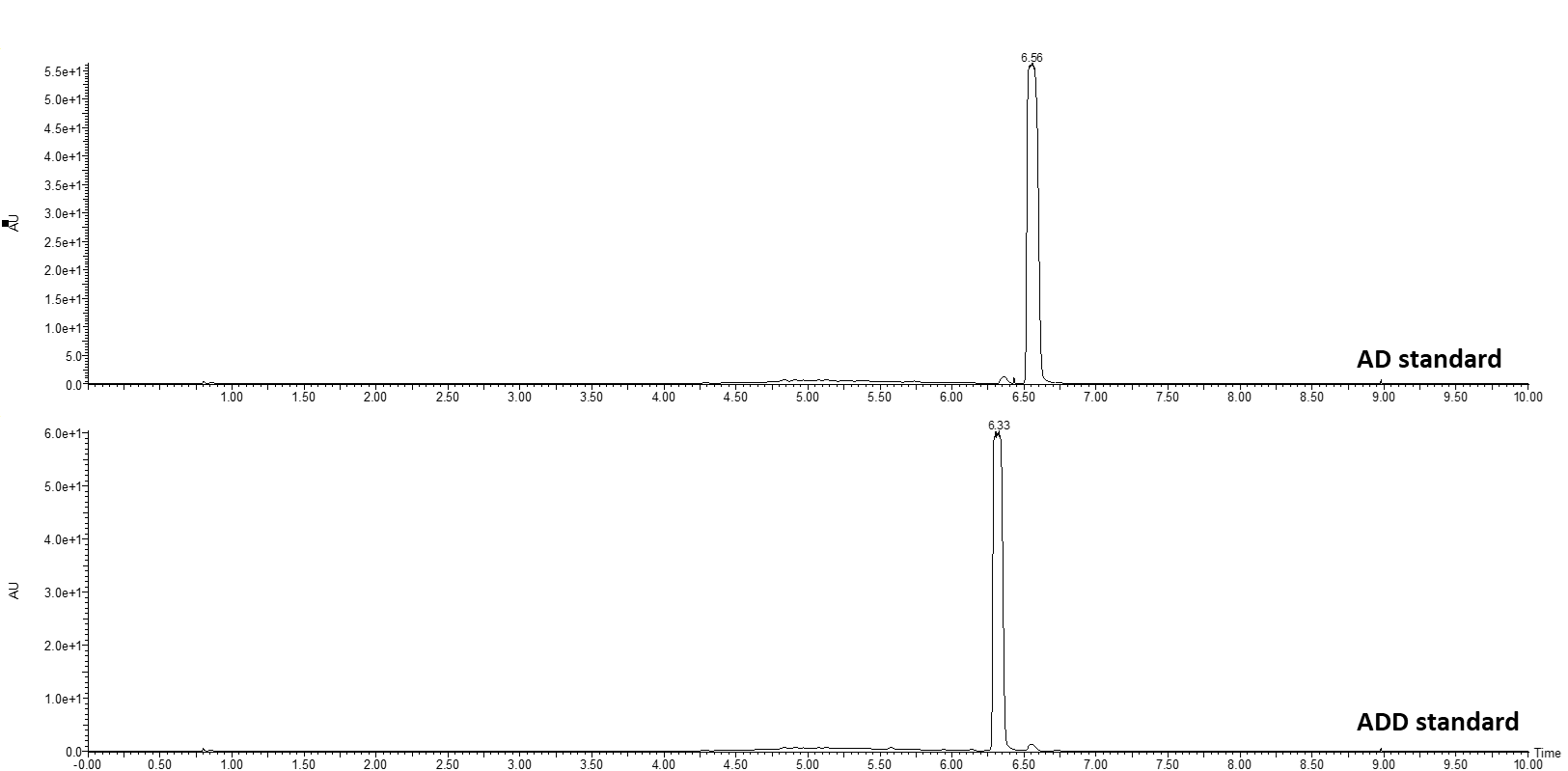


**B.**

**Supplementary Table 1.** Plasmids used in this work

| Plasmid name | Type | Use |
| --- | --- | --- |
| pJV53 | Replicative/ Kn^R^ | Study of transformation with replicative vectors |
| pMC1s | Integrative/ Kn^R^ | Study of transformation with integrative vectors at *att*B from L5 phage |
| pMY769 | Integrative / Str^R^ | Study of transformation with integrative vectors at *att*B from L5 phage |
| pTTP1B | Integrative / Kn^R^ | Study of transformation with integrative vectors at *att*B from Tweety phage |
| pMH3 | Suicide / Kn^R^ | Vector to generate single recombination mutants in *Ml .hassiacum*. |
| pMH3::*kstD4* | Suicide/ Kn^R^ | Obtaining of *kstD*_KO mutants by single homology recombination |

**Supplementary Table 2.** Primers used in this work. Underlined sequences corresponds to

restriction sites included in the sequences

| Primer name | Sequence 5’>3´ | Use |
| --- | --- | --- |
| Prom_pTTP1B_Fw | TTTGACGTCTCCTGGTATGCAGCCT | PCR to amplify promoter from pTTP1B |
| Prom_pTTP1B_Rv | CATATGAACACCCCTTGTATTACT | PCR to amplify promoter from pTTP1B |
| kstD_Fw | AAACTCGAGATGCTGTCGTTCGTCAT | PCR amplification of internal *kstD4* fragment |
| kstD_Rv | TTTCTCGAGGATCACCGATCCGTCGT | PCR amplification of internal *kstD4* fragment |
| kstD_KO_Fw1 | AAGCTCGACATCATGGAG | PCR confirmation of *kstD*4 mutants |
| kstD_KO_Rv1 | ACACAGGAAACAGCTATGAC | PCR confirmation of *kstD*4 mutants |
| kstD_KO_Fw2 | AGAAACTTCTGGAATCGCTAGAG | PCR confirmation of *kstD*4 mutants |
| kstD_KO_Rv2 | ATGGCGTTTGAGCTGGTTG | PCR confirmation of *kstD*4 mutants |
| kstD_KO_Fw3 | TGCGCTGACATTGCCT | PCR confirmation of *kstD*4 mutants |
| kstD_KO_Rv3 | AGAAGATCGACACCTACCTCGAA | PCR confirmation of *kstD*4 mutants |
